# Epigenetics in Traditional Chinese Pharmacy: A Bioinformatic Study at Pharmacopoeia Scale

**DOI:** 10.1093/ecam/neq050

**Published:** 2011-03-08

**Authors:** Hsin-Ying Hsieh, Pei-Hsun Chiu, Sun-Chong Wang

**Affiliations:** ^1^Institute of Systems Biology and Bioinformatics, National Central University, Chungli, Taoyuan, Taiwan, China; ^2^Epigenetics Laboratory, Centre for Addiction and Mental Health, Toronto, Ontario, Canada

## Abstract

Epigenetics is a phenomenon of heritable changes in the chromatin structure of a genomic region, resulting in a transcriptional silent or active state of the region over cell mitosis. Mounting evidence has demonstrated phenotypic consequence of alternations in the patterns of DNA methylation and histone modifications, two of the well-studied epigenetic mechanisms. The epigenome thus represents an interesting therapeutic target. Traditional Chinese medicine (TCM) is a system of therapies that has developed through empiricism for over 2100 years and has remained a popular alternative medicine in some Far East Asian populations. We searched 3294 TCM medicinals (TCMMs) containing 48 491 chemicals for chemicals that interact with the epigenetics-related proteins and found that 29.8% of the TCMMs are epigenome- and miRNA-modulating via, mainly, interactions with Polycomb group and methyl CpG-binding proteins. We analyzed 200 government-approved TCM formulas (TCMFs) and found that a statistically significant proportion (99%) of them are epigenome- and miRNA-interacting. The epigenome and miRNA interactivity of the Monarch medicinals is found to be most prominent. Histone modifications are heavily exploited by the TCMFs, many of which are tonic. Furthermore, epigenetically, the Assistant medicinals least resemble the Monarch. We quantified the role of epigenetics in TCM prescription and found that epigenome- and miRNA-interaction information alone determined, to an extent of 20%, the clinical application areas of the TCMFs. Our results provide (i) a further support for the notion of the epigenomes as a drug target and (ii) a new set of tools for the design of TCM prescriptions.

## 1. Introduction

According to a recent survey, 40% US adults had at least once used alternative and complementary medicine (CAM) therapy during a 1-year span [1]. The prevalence of CAM among adults >65 years old was even higher at 63% [2]. Traditional Chinese medicine (TCM) is a system of theories and therapies that was first documented in ancient Chinese classics dating back 2100 years [3]. It has since undergone substantial development and improvement under the auspices of ruling authorities as well as private practitioners in China [4, 5]. TCM survives the displacement of modern Western medicine and continues to serve as one of the major CAMs among Far East Asian populations nowadays. In particular, in China, Japan, Korea and Taiwan, standardized TCM formulas (TCMFs) have been approved by the health regulatory agencies and thus covered by the public health insurance. TCM utilizes natural products, acupuncture, chiropractic and a combination of them in a therapeutic course. We discuss only the TCM usage of natural products that have known chemical compositions in this article.

TCM ascribes an individual's ailment to an imbalance in the antagonizing elements in a part (or parts) of the individual's body [3, 6–9]. The opposing forces are broadly called *yin* and *yang*. A TCM practitioner differentiates a patient's syndromes into different categories of *yin* or *yang* deficit. TCM medicinals (TCMMs), which are characterized into *yin*- or *yang*-enhancing categories, are then prescribed as a cure to restore the *yin-yang* balance [10]. The intangible *yin-yang* and other differentiations, such as emptiness-fullness and coolness-warmness, of a patient and the associated TCM treatments reflect in part the limitations of the diagnostic and clinical techniques of the ancient East in comparison with the advances in biochemistry and molecular biology of the modern West. Sustainability and renaissance of TCM call for a modernization of TCM, where standardization of TCM terminologies, materials and formulas is seen as among the first endeavors [11, 12]. Our approach toward the goal considers elucidating epigenetic mechanisms in TCMMs and TCMFs. The latter are composed of combinations of the former and serve as the major form of TCM prescriptions a patient receives [13, 14].

Epigenetics refers to mitotically stable changes in gene expression that do not involve alterations in the underlying DNA sequence. Well-known epigenetic mechanisms in mammalian cells include methylation of CG dinucleotides, post-translational modifications of histone proteins and RNA interference [15, 16]. For example, DNA methylation, histone deacetylation and microRNA (miRNA) expression are associated with gene silencing [17, 18]. The profile of epigenetic marks (epigenome) and miRNA expression of a cell is sensitive to environmental perturbations [19, 20] and aberrations in the profile can lead to an increased predisposition to morbid phenotypes such as Alzheimer's disease [21], autoimmune disease [22] and heart disease [23]. In particular, a genome-wide hypomethylation and gene-specific hypermethylation are considered a hallmark of cancerous cells [24]. Many TCM prescriptions are believed to exert a long-lasting change to an individual's constitution. Furthermore, TCM prescriptions are thought to lack target specificity in contrast to the particular receptor/pathway targeting of modern Western drugs. The former property of TCM is reminiscent of the maintenance of (acquired) epigenetic patterns through somatic cell divisions and the latter of (i) the epigenetic (dys)homeostasis affecting the entire epigenome and (ii) single miRNA targeting different mRNA sequences. The correspondences motivated our hypothesis of an epigenetic role in the pharmacology of TCM prescriptions.

The hypothesis was addressed in a systematic way by interrogating a large collection of TCMMs and TCMFs, which has become achievable in the era of data explosion and open access. Namely, we obtained the information about direct interactions between chemicals and epigenetics-related enzymes from a chemical-protein association database [25], which contains 10 288 993 pairs of chemical-protein interactions. We retrieved the information about the chemicals in TCMMs from two TCMM databases [26, 27], which together contain 48 491 chemicals in 3294 TCMMs. We obtained, from a government database [28], the compositions of 200 standardized TCMFs, the prescriptions of which are reimbursed under the public health insurance in Taiwan.

The human proteins of our attention are known to be involved in epigenetic regulation and believed to be expressed in most cell types. They include DNA methyltransferase (DNMT), histone deacetylase (HDAC), histone acetyltransferase (HAT), histone methyltransferase (HMT), methyl CpG-binding protein (MBD), Polycomb group protein (PcG), Drosha and Dicer (DICER) and their families. The former four catalyze covalent modifications of chromatin. MBDs and PcGs (co)localize in heterochromatic loci. DICER process miRNA precursors to mature miRNA. Evidence of a TCMM's modulation of the epigenome and miRNA was then sought through its interactions with the human proteins. Characterization of a TCMM, that is, taxonomical kingdom, TCM nature, TCM flavor and TCM meridian, is of interest as the characteristics serve as a guide for its functions [3, 6]. We identified the overrepresented characteristics of the epigenome- and miRNA-interacting TCMMs. A TCMM, based on its role in the formula, can assume one of the four positions: (i) Monarch targeting the main syndrome, (ii) Minister strengthening the effect of the Monarch or addressing the secondary syndromes of the patient, (iii) Assistant enhancing the curative effect of the Monarch and Minister or allaying the drastic and toxic effect of the Monarch and Minister and (iv) Guide harmonizing or leading the prescription to the offending part of the body [3, 6]. We investigated if different epigenetic mechanisms are utilized by the TCMMs at different positions. We studied if a TCMF's epigenome and miRNA interactivity (over the TCM positions or epigenetic mechanisms) differentiates its area of clinical applications. The degree of successful differentiation helped provide a quantification of the role of epigenetics in TCM prescription.

## 2. Methods

### 2.1. Chemical-Protein Interactions and TCMMs Databases

The chemical-protein interactions were obtained from the STITCH database (http://stitch.embl.de/) [25], containing 10 288 993 pairs of chemical and protein associations. Each pair of association comes with a score for the degree of evidence for the association. The evidence score ranges between 999 and 150 for the types of evidence from direct experimental evidence of chemical-protein binding, manual annotation of metabolite–protein reactions, chemical-protein interactions across species, to MEDLINE and OMIM text mining [25]. Information on TCMMs is mainly from the government-backed Shanghai TCM Data Center (STDC) (http://www.tcm120.com/1w2k/tcm_species.asp) [26] containing information about the natures, flavors, meridians and chemicals of 8896 TCMMs. We also incorporated the chemicals data of 50 TCMMs from TCM-ID (http://tcm.cz3.nus.edu.sg/group/tcm-id/tcmid.asp) [27], which contains 1313 TCMMs, because the 50 medicinals have more chemical entries in TCM-ID than in STDC. The TCMFs were from the Department of Health, Taiwan (http://www.ccmp.gov.tw/en/information/formula.asp) [28], which lists 200 standardized TCMFs with the composing medicinals and their weights in grams as well as the dose of the formula per day in grams. The weights of the medicinals in the formulas range from 0.15 to 25 g with a mean (median) of 3.5 (3.0) g. The 200 daily doses range from 21 to 39 g with a mean (median) of 28.4 (28.0) g.

The chemicals in a TCMM are the very information for us to establish its epigenome- and miRNA-interactivity. Supplementary Figure S1 shows the distribution of the numbers of chemicals in the TCMMs from the integrated STDC/TCM-ID database, the median number of chemicals per TCMM being nine. The CAS registry numbers in STDC and TCM-ID were translated to PubChem IDs, which are used in STITCH, using the PubChem server (http://pubchem.ncbi.nlm.nih.gov/).

The 200 TCMFs are made from a total of only 276 different TCMMs. Supplementary Figure S2 shows the distribution of the numbers of medicinals per formula, the median number being eight. (Note that only 230 of them were unambiguously and non-redundantly mapped to the integrated STDC/TCM-ID database due to different naming of TCMM parts/preparations between the systems.) Some TCMMs are common among the formulas. Supplementary Figure S3 shows the frequency of medicinals in the 200 formulas. Designation of the formulas to the clinic function categories is available from the Bureau of National Health Insurance, Taiwan (http://www.nhi.gov.tw/). Supplementary Figure S4 shows the distribution of the functional categories of the 200 formulas.

### 2.2. Data Analysis

#### 2.2.1. Determination of Epigenetic TCMMs

A medicinal is DNMT-interacting if it contains chemicals that interact with the DNMT family proteins, which were identified from a search with keywords “DNMT" in the STITCH protein annotation file (protein.aliases.v7.1.txt). A medicinal can contain more than one DNMT-interacting chemical, each of which may interact with more than one proteins in the DNMT protein family. The strength of DNMT interaction of the medicinal was determined by the total number of such interactions multiplied by the sum of average evidence score per chemical divided by the total number of DNMT-interacting chemicals in the STITCH database. The multiplication is to weight by the evidence. The division is to account for the fact that some proteins contain more pairs of interactions because they are better studied. Procedures for other epigenetic mechanisms were similar with HDAC identified with keywords “histone" and “deacetylase", HAT with keywords “histone" and “acetyltransferase", HMT with “histone" and “methyltransferase", MBD with “methyl", “CpG" and “binding", PcG with “polycomb", and DICER with “dicer" and “drosha". The matchings were all case-insensitive. The numbers of proteins thus identified in each of the DNMT, HDAC, HAT, HMT, MBD, PcG, DICER families are respectively 6, 12, 7, 8, 9, 8, 1 and 1. They are listed in Supplementary Table S1. A medicinal is called epigenetic if it is X-interacting where X is any of the seven epigenetic mechanisms. The epigenome- and miRNA-interactivity of the medicinal is the sum of the seven interaction strengths.

#### 2.2.2. Determination of Epigenetic TCMFs

A formula is epigenetic if at least one of its composing medicinal is epigenetic. The X-interactivity of the formula is obtained from the sum of strengths of X interaction of the composing medicinals multiplied by the grams of the medicinals in the formula.

Further analyses, including determination of a medicinal's TCM position in a formula, are given in online Supplementary Data.

## 3. Results

As we are studying the roles of epigenetics in TCM through chemical-protein interactions, we focus on the TCMMs that hold non-zero chemical entries in the databases. After combination of two databases, the number of such TCMMs is 3294, containing a total of 48 491 chemicals, over 66% of which are mapped to PubChem. The numbers are the largest in such materia medica-level studies to our knowledge. Among the TCMMs, 3284 have taxonomy annotations, in which 90% are plants, 9% are animals and 1% are minerals. The proportions are not close to those (69, 18, 13 and 58, 23, 19%) in the classical TCM literature *Shen Nong Ben Cao Jing* [6] and *Ben Cao Gang Mu* [29], which contain 365 and 1892 TCMMs, respectively. The discrepancy may reflect shifts in TCMM usage away from extinct animals and toxic minerals toward more diverse and accessible plants.

### 3.1. Specificity of TCMMs to Epigenetic Mechanisms

**Figure 1:**
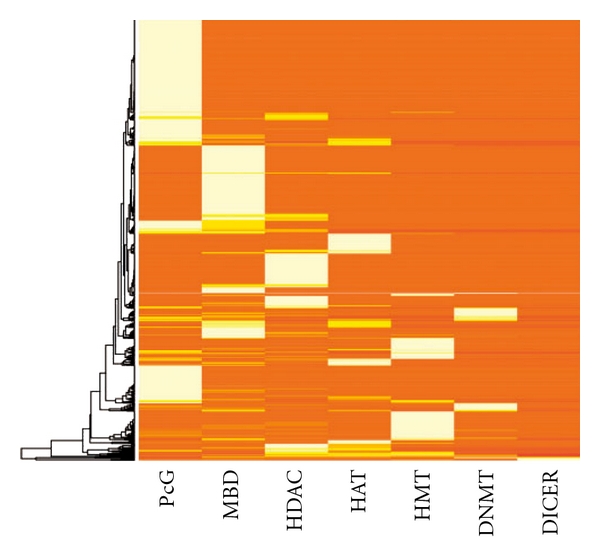
Epigenome- and miRNA-interactivity of the 981 epigenetic TCMMs over epigenetic mechanisms. A row represents a medicinal. Lighter shades indicate stronger interactivity. Ordering of the rows was by unsupervised hierarchical clustering. Columns were ordered according to the prevalence of the mechanism among the 981 epigenetic TCMMs. Note that DICER includes Drosha and Dicer in the RNAi pathway.

A TCMM is epigenetic, if at least a chemical in it shows evidence of direct interaction with any of the epigenetics-related proteins. We found that 29.8% or 981 out of the 3294 TCMMs are epigenetic. In specific, a TCMM is X-interacting if at least a chemical in it shows evidence of direct interaction with proteins X. We found, in Figure 1, the distribution of the operating mechanisms among the 981 epigenetic TCMMs (epiTCMMs), there being 480 PcG-interacting, 384 MBD-interacting, 260 HDAC-interacting, 162 HAT-interacting, 149 HMT-interacting, 71 DNMT-interacting and 17 DICER-interacting epiTCMMs. As Figure 1 shows, many epiTCMMs operate through only one or few (1.6 ± 1.0), but not all, of the mechanisms under study, suggesting specificity of TCMMs to particular epigenetic mechanisms.

**Figure 2:**
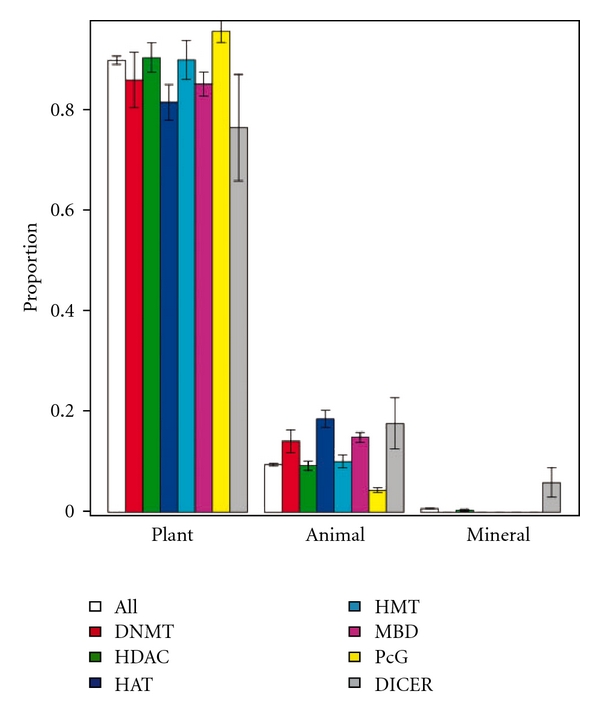
Kingdom distribution of mechanism-specific epigenetic TCMMs. In total, 3284 TCMMs have kingdom annotation. Among them 980 are epigenetic. More PcG-interacting TCMMs originate from plants than expected (*P* = 4.2 × 10^−7^). More MBD- and HAT-interacting medicinals are from animals than expected (*P* = 8.4 × 10^−5^ and 1.1 × 10^−4^, resp.).

Supplementary Figure S5 plots the proportions of TCMMs originating from plant, animal and mineral sources. An overrepresentation analysis (Supplementary Data) indicated that, given the proportions of all the TCMMs in plants, animals and minerals, epiTCMMs appear slightly more in plants than expected (*P* = .041). Furthermore, when stratifying the epiTCMMs according to the operating mechanism, we found, in Figure 2, that PcG-interacting TCMMs are overrepresented in plants (*P* = 4.2 × 10^−7^) and MBD- and HAT-interacting TCMMs in animals (*P* = 8.4 × 10^−5^ and 1.1 × 10^−4^, resp.).

### 3.2. Specificity of Epigenetic TCMMs to TCM Natures, Flavors and Meridians

**Figure 3:**
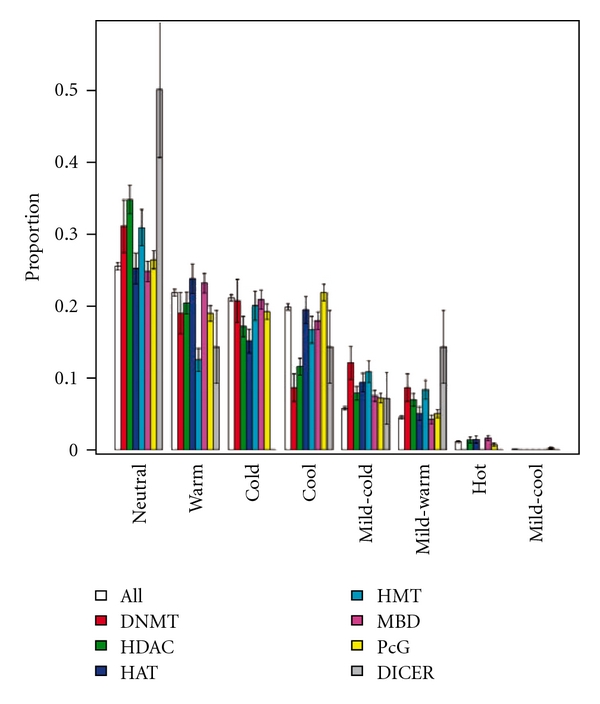
Nature distribution of mechanism-specific epigenetic TCMMs. In total, 2530 TCMMs have TCM nature annotation. Among them 818 are epigenetic. At a *P*-value cutoff of 0.01, HDAC-interacting TCMMs are overrepresented in neutral (*P* = .00041).

**Figure 4:**
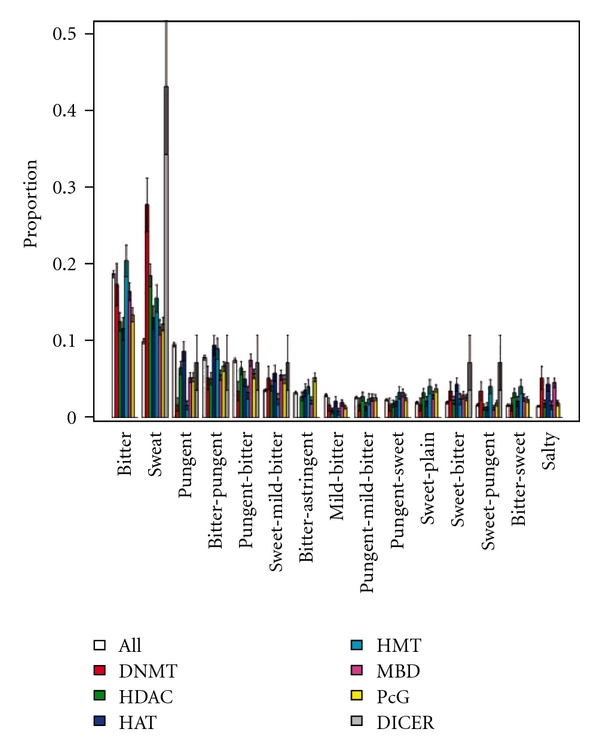
Top 15 flavor distribution of mechanism-specific epigenetic TCMMs. In total, 2540 TCMMs have flavor annotation. Among them 822 are epigenetic. DNMT-interacting TCMMs are overrepresented in sweet (*P* = 7.1 × 10^−5^); HDAC-interacting medicinals in sweet, sweet-sour and sour-sweet (*P* = 2.4 × 10^−5^, 7.5 × 10^−5^ and 1.7 × 10^−3^, resp.); HAT-interacting medicinals in bitter-sweet-astringent (*P =* 8.4 × 10^−3^); MBD-interacting medicinals in salty (*P* = 7.4 × 10^−5^); PcG-interacting medicinals in sweet-plain and bitter-astringent (*P* = 3.8 × 10^−3^ and 6.7 × 10^−3^); and DICER-interacting medicinals in sweet (*P* = 1.2 × 10^−3^). The above overrepresentations are for *P* < .01.

**Figure 5:**
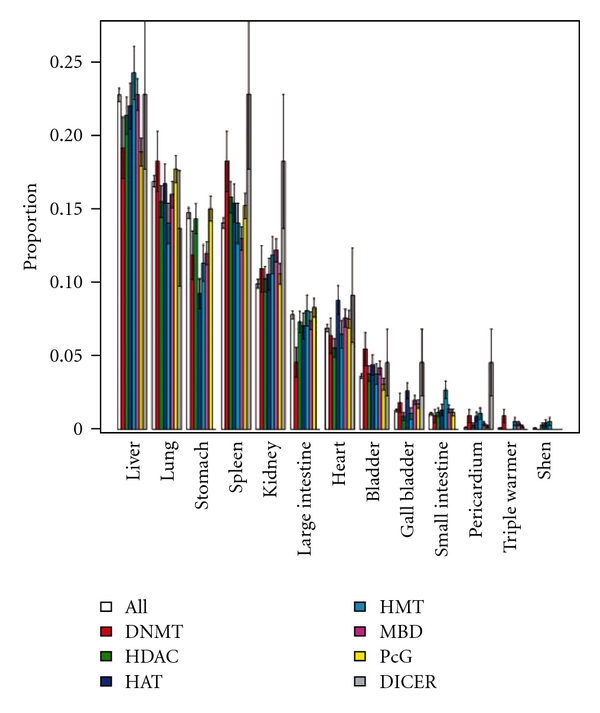
Meridian distribution of mechanism-specific epigenetic TCMMs. In total, 1155 TCMMs have meridian annotation. Among them 452 are epigenetic. Note that unlike natures and flavors, a TCMM can possess more than one meridians. DNMT- and HDAC-interacting TCMMs are overrepresented in spleen (*P* = .047 and .039, resp.); HAT-interacting medicinals in pericardium and gall bladder (*P* = .034 and .046, resp.); HMT-interacting medicinals in pericardium and small intestine (*P* = .024 and .030, resp.); MBD-interacting medicinals in kidney and liver (*P* = .011 and .047, resp.); PcG-interacting medicinals in spleen and lung (*P* = .038 and .044, resp.); and DICER-interacting medicinals in pericardium (*P* = .031). The above overrepresentations are from *P* < .05.

TCMMs are characterized according to TCM theories to different TCM natures and flavors [3, 6]. Supplementary Figures S6 and S7 show the nature and flavor distributions of the TCMMs. Similar to the taxonomy overrepresentation analysis, *sweet* epiTCMMs are found more often than they are expected (*P* = .0071) at a *P*-cutoff of 0.01. A TCMM is also designated its targeting TCM meridians [29], whose distribution is shown in Supplementary Figure S8. The overrepresentation analysis indicated that epiTCMMs target more often kidney and spleen meridians than expected (*P* = .020 and .041, resp.). We also further differentiated the epiTCMMs by their operating epigenetic mechanisms. Results of the mechanism-specific overrepresented epiTCMMs are shown in Figures 3, 4, and 5.

### 3.3. Overrepresentation of Epigenetic TCMFs

Clinically relevant TCM prescriptions are in the form of formulas composed of multiple TCMMs. The role of epigenetics in TCM is therefore best tested by examining the involvement of epigenetics in TCMFs. A TCMF is called epigenetic if at least one of the composing TCMMs is epigenetic. Among the 200 standardized TCMFs, 198 were found to be epigenetic. In Supplementary Data, we calculated the probability, *P*, of getting 198 epigenetic TCMFs (epiTCMFs) if the 200 TCMFs were formed by randomly combining TCMMs from the pool of TCMMs that form the TCMFs and found *P* = 2.2 × 10^−5^. The small *P*-value lends support for the proposition of an epigenetic role in traditional Chinese pharmacy.

**Figure 6:**
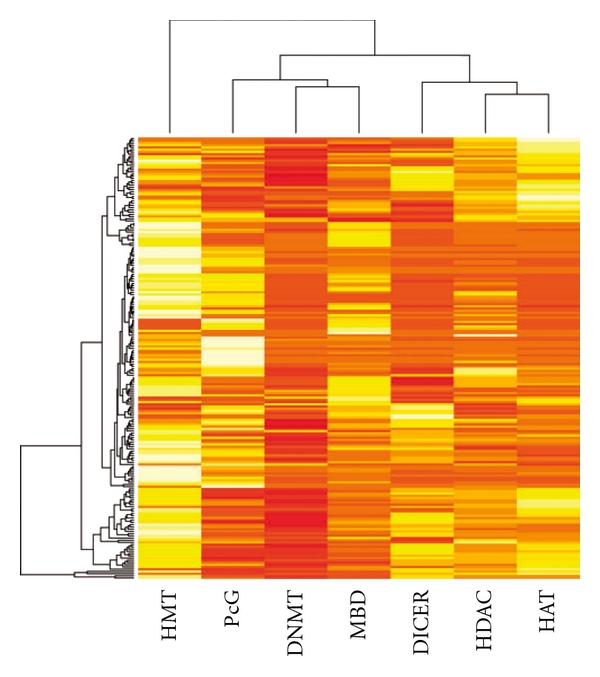
Epigenome- and miRNA-interactivity of the 198 epigenetic TCMFs over epigenetic mechanisms. A row represents a TCMF and a column a mechanism. Rows and columns are ordered by the unsupervised hierarchical clustering algorithm. Lighter shades indicate stronger interactivity.

Again, if we distinguish the epiTCMFs according to the mechanism(s) they operate, the pattern of epigenetic mechanisms in TCMFs is revealed. As Figure 6 shows, unlike epiTCMMs, an epiTCMF operates via more than one (5.5 ± 2) mechanisms in delivering the epigenome- and miRNA-interactivity, reflecting the fact that a TCMF is made up of multiple TCMMs. Figure 6 also shows that histone modifications are among the top working mechanisms in the 198 epiTCMFs. Supplementary Figure S9 sums the degree of involvement of each mechanism over the 198 epiTCMFs. Furthermore, the clustering of HAT, HDAC, DICER and of MBD, DNMT and PcG in Figure 6 provide a tempting hint at the synergy of epigenetic mechanisms in TCMFs.

### 3.4. Specificity of Epigenome-Interactivity to TCM Positions

**Figure 7:**
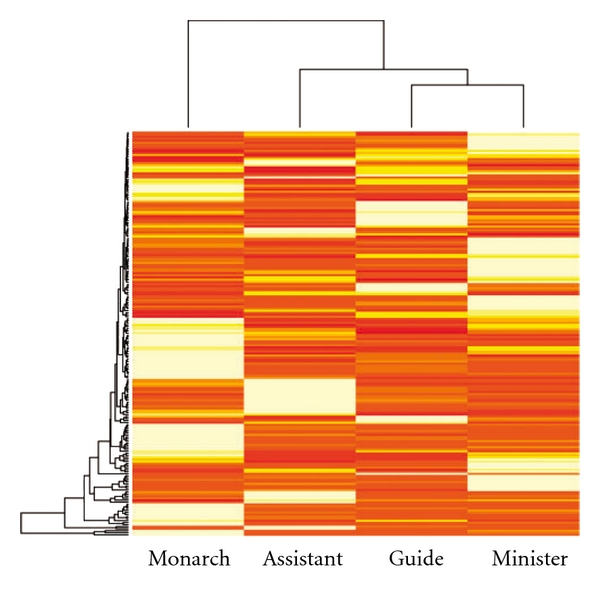
Epigenome- and miRNA-interactivity of the 198 epigenetic TCMFs over TCM positions. Rows represent TCMFs and columns the four TCM positions. The lighter the shades the stronger the interactivity. Orders of both rows and columns are arranged by the unsupervised hierarchical clustering algorithm. Monarch medicinals are distinct from the other positions and least similar to Assistant.

**Figure 8:**
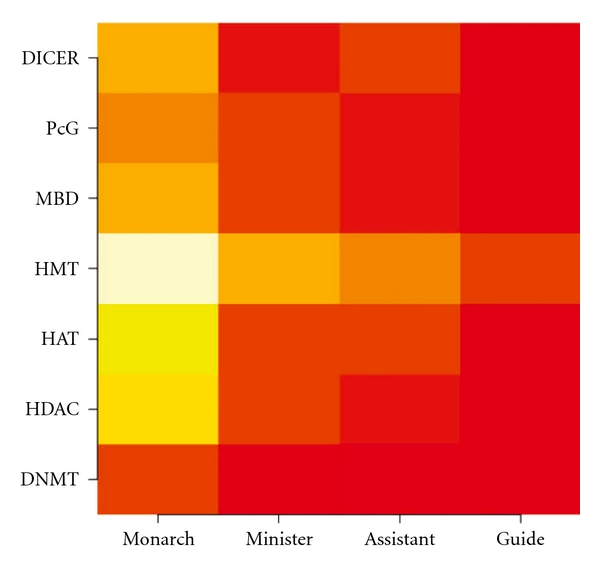
Pattern of utilization of epigenetic mechanisms over TCM positions. Medicinals in a formula can be differentiated by their positions in the formula into Monarch, Minister, Assistant and Guide. The mechanism-specific interactivity of the Monarch (and other) medicinals in the 198 epigenetic TCMFs is calculated and summed. The lighter the shades, the larger the interactivity.

In formula design, medicinals are chosen to take the four positions, Monarch, Minister, Assistant and Guide, according to the principles of TCM prescription [3, 6]. The role of a medicinal in a formula therefore can be understood by its position. We identified the position of each medicinal in the 200 formulas and calculated the epigenome- and miRNA-interactivity of the medicinals occupying the same position (Supplementary Data). The result in Figures 7 and 8 shows that Monarch medicinals are epigenetically distinct in that Monarch are most epigenome- and miRNA-interacting, followed by Minister, Assistant and Guide. Furthermore, Monarch and Assistant medicinals are found epigenetically least similar among the possible pair-wise comparisons (Supplementary Table S2).

Since the interactivity was calculated to be proportional to the degree of evidence of chemical-protein interactions defined in [25] (see “Methods" section), we repeated the analysis using the numbers of epigenetic mechanisms a medicinal possesses, instead of the numbers of epigenome- and miRNA-interacting chemicals and the associated evidence score, in the calculation of interactivity. Supplementary Figures S10a, S11a and Table S2 show the result and the conclusion that Monarch is epigenetically distinct and least similar to Assistant remains. Since, according to the principles of TCM prescription, Monarch medicinals are the major medicinals against the syndrome and many Assistant medicinals are antagonistic to the Monarch in order to rein in the toxicity or side-effects of the Monarch, the results of an epigenetic uniqueness of Monarch and epigenetic disparity between Monarch and Assistant may be considered a demonstration of the principles of TCM prescription.  Figure 8 unfolds the epigenome-and miRNA-interactivity of the TCMFs over the mechanisms to reveal heavy exploitation of histone modifications (cf Figure 6 and Supplementary Figure S9) by the Monarch. However, the exploitation shifts to MBD and PcG interactions when scores are not taken into account as shown in Supplementary Figure S11a. Results of the analysis at the two extreme settings suggest prioritizing histone modifications and PcG interaction for experimental verification of TCMF epigenetics.

### 3.5. Quantification of Epigenetic Role in TCMFs

A functional categorization of TCMFs according to the areas of TCM clinical applications has been developed and widely adopted since 1682 [30]. Supplementary Figure S4 plots the distribution of the 200 standardized TCMFs across 20 categories and shows that tonic formulas account for most of the formulas, followed by fire-purging formulas and dampness-eliminating formulas, and so forth.

**Figure 9:**
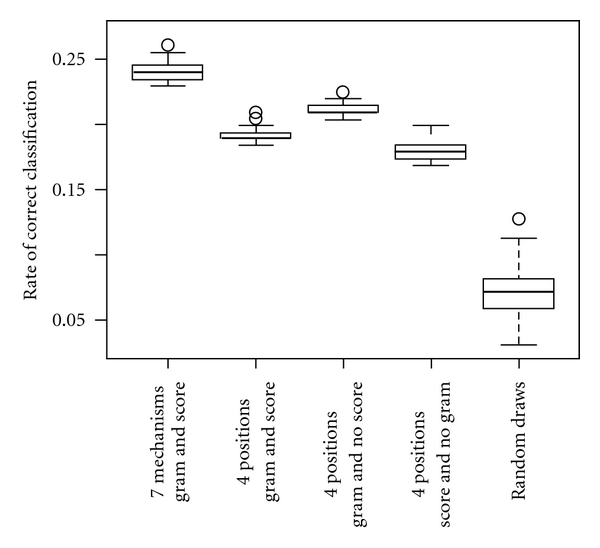
Rate of successful functional categorization of TCMFs by epigenome- and miRNA-interactivity. TCMFs were classified by the mechanism-wise (first column) or position-wise (second, third and fourth columns) epigenome- and miRNA-interactivity. In the position-wise studies, evidence scores of chemical-protein interactions (doses of medicinals) were not utilized in the third (fourth) column. The fifth column is the rate from random guess based on the proportion of categories among the formulas.

In an effort to quantifying the role of epigenetics in TCM prescription, our approach lies in answering the question of how accurately we can determine the functional category of a formula based solely on its epigenome- and miRNA-interactivity. To the end, we employed a cross validation scheme detailed in Supplementary Data. The result in Figure 9 indicates that epigenetic consideration plays a sizable role, 19 ± 1%, in prescribing TCMFs for the various syndromic categories. To assess the significance, we estimated the success rate when the category determination was randomly made based on the proportion of the functional categories among the 200 TCMFs. The rate of success by such random draws was found to be 7 ± 2% (Supplementary Data), which is significantly lower than that by the epigenetic information. Furthermore, Figure 9 indicates that information of the epigenome- and miRNA-interactions at the four TCM positions, without the evidence score, achieved a comparable success rate of 21 ± 1%. The result may illustrate the essential role of positions in formula formation. Note that although the success rate by mechanism-specific epigenetic information is higher at 24 ± 1%, its application may not be as handy as the four position method. The method of TCMF categorization via epigenome and miRNA interactivity may help in the design of new formulas. An example is provided in “Discussion" section.

## 4. Discussion

Epigenetic phenomena have been recognized as a crucial component in the normal development of eukaryotes including plants [31] and animals [32]. It thus may not be surprising to find that many, that is, 29.8% of the 3294 TCMMs, most of which are derived from plants and animals, are potentially affecting the epigenomes and miRNA expression of human cells. We chose the proteins for their well characterization and documentation [33, 34], which helped support our comprehension of TCMM epigenetics. For example, eight of the HDAC/HAT-modulating plant sources (i.e., cashew, licorice, celery, apple, tea, onion, peanuts and red grapes) reported in the review [35] are included in our 3294 TCMM collection. Among them, all except one (i.e., cashew) were identified to be HDAC- or HAT-interacting through our chemical-protein association approach. Furthermore, four dietary sources (i.e., tea, soy, cabbage and turnip) reported to be DNA methylation modifying in [36] are also in the collection and two of them (i.e., tea and turnip) were by our approach identified to be MBD-interacting, where MBDs were known to interact with DNMTs [37]. In Supplementary Tables S3–S9, we list all the identified epigenetic TCMMs. The tables may serve as a priority list for future experimental verification of TCMM epigenetics.

We identified the taxonomic kingdoms and TCM natures, flavors and meridians that accommodate more epiTCMMs than expected (Supplementary Figures S5–S8). In particular, Figures 2, 3, 4, and 5 show the mechanism-specific epiTCMMs that are overrepresented. A recent study reported an essential requirement for O-GlcNAc glycosylation of Polycomb proteins to maintain transcriptional repression in *Drosophila* [38]. The result in Figure 4 of overrepresented PcG-interacting TCMMs in sweet-plain flavor may be a manifestation of such a connection between glycosylation and PcG function. More studies are needed in this regard.

Our epigenetic analysis of the TCMFs over the positions revealed a decreasing interactivity from Monarch to Guide. Since medicinal doses generally decrease gradually from Monarch to Guide, we repeated the analysis by setting all the grams to 1. The result, shown in Supplementary Figures S10b, S11b and Table S2, upholds the finding that Monarch medicinals heavily exploit epigenetic mechanisms, followed by Minister, Assistant and Guide, and that Monarch and Assistant are epigenetically least similar. In the perspective of TCM epigenetics, Minister, Assistant and Guide serve to fine tune the epigenome- and miRNA-interactivity of the prescription, making multi-medicinal formulas a better treatment to patients than single medicinals in some cases. Since medicinal doses in a formula are an essential tuning parameter for the formula's function according to the principles of TCM prescription, on comparing the success rate of functional prediction with gram information (second column of Figure 9) to that without grams (fourth column of Figure 9), indeed we found that the former 19 ± 1% is slightly higher than the latter 18 ± 1%, consistent with the principles of TCM prescription. The slight improvement with grams however may reflect the fact that a medicinal's position determines its dose. Medicinal dosage therefore could become redundant given its position.

**Figure 10:**
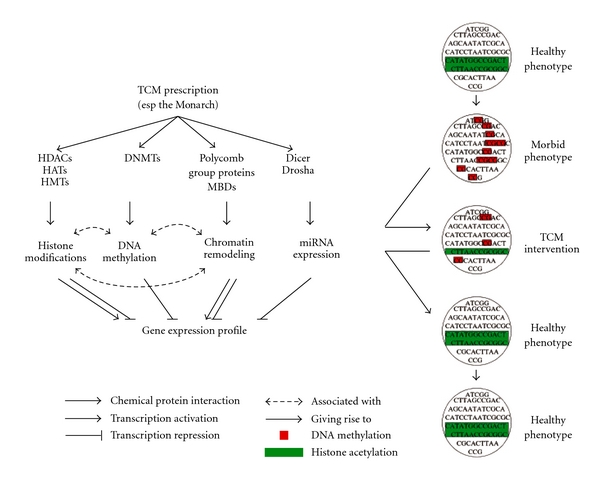
A model of the holistic and long-term therapeutic effect of TCM prescriptions. Patterns of epigenetic marks such as DNA methylation (red) and histone acetylation (green) help maintain the gene expression profile for normal cell functioning. Aberrations in the epigenetic pattern, due to endogenous or exogenous factors, lead to disorders. Compounds in TCMFs, in particular the Monarch, interact with the epigenetics-related proteins to restore the altered epigenetic pattern, curing the disorder. Since the epigenetics-related proteins potentially affect the whole epigenome and the epigenetic pattern, once restored, is passed on to the daughter cells, the holistic (in contrast to reductionist's single gene or pathway) and long-term effects of TCM prescriptions are realized.

As we and colleagues recently demonstrated that epigenetic aberrations play an important role in neurobehavioral diseases such as schizophrenia, bipolar disorder [39] and Alzheimer's disease [21], the epigenomes may represent a promising target for drug development. Indeed, as we found that most of the 200 basic TCMFs are potentially epigenome- and miRNA-modulating and since many TCMFs have, on an empirical ground, been considered efficacious, our finding further argues for the epigenomes as a candidate for drug target. As the targets of the epigenetics-related proteins are multiple and the acquired epigenetic patterns are mitotically stable, the holistic and long-term effects of TCM prescriptions can be attributed to TCM epigenetics. We summarize in Figure 10 the proposed model of action of TCM prescriptions.

As an example of the applications of TCM epigenetics, we consider a TCMF that was recently prescribed, as a complementary to the routine Western medicine treatment, for SARS-like pandemic [40]. The formula consists of 13 medicinals, the position-wise epigenome- and miRNA-interactivity of which was straightforwardly calculated (Supplementary Data). By comparing with those of the 200 TCMFs, we conclude that the SARS-TCMF prescription is epigenetically a fire-purging formula. As one of the main symptoms of SARS is high fever over 38°C and the combined treatment was shown in the preliminary study to shorten the days toward an improvement (in the chest radiography) [40], we may propose to attribute the beneficial effect of the combined treatment to the TCMF's fire-purging function. Supplementary Tables S3–S9, which list all the epiTCMMs identified in the present article, therefore may serve to help prescribe formulas for emerging syndromes.

A TCMF comprises a number of TCMMs. Moreover, co-prescription of a TCMF with a TCMM (or another TCMF) is not uncommon and in fact has been on a rise clinically [41], possibly responding to the condition of multiple co-morbidities due to such phenomena as gene–gene interactions. Further studies on prescriptions combining TCMFs and TCMMs are needed. However, like other approaches, our current bioinformatics study is not short of limitations. Since many TCMFs are served in the form of decoctions, whether the epigenome- and miRNA-modulating chemicals survive the mixing and processing is unclear. After digestion, whether the chemicals reach the nuclei of the targeted cells is not clear. Moreover, missing information about the direction of action (activation or inhibition) of chemical-protein interactions hindered a discussion of the relations among the various epigenetic mechanisms in the TCMFs. However, even although only 30% of the TCMMs are epigenetic, 99% of the TCMFs are found epigenetic, through mainly the Monarch, which are known to be the major medicinals in a TCM prescription. Such a large, pharmacopeial scale analysis of epigenetics in TCM materials offers a strong support for the proposition of an epigenetic role in TCM pharmacology. Epigenetics of TCM may provide a new land for TCM research.

## Supplementary Data

Supplementary data are available at *eCAM* online.

## Funding

This work was supported in part by National Science Council, Taiwan [99-2112-M-008-003-MY3].

## Supplementary Material

Further analysis, supplementary figures, table of epigenetic proteins, tables of potentially epigenome-interacting TCM medicinal.
