# Supplementary material for: Epigenetics in Traditional Chinese Pharmacy: A Bioinformatic Study at Pharmacopoeia Scale
Source: Evid Based Complement Alternat Med. 2011 Mar 8;2011:816714. doi: 10.1093/ecam/neq050 (PMC3137654; doi:10.1093/ecam/neq050)
Supplement: Supplementary file 1 — Further analysis, supplementary figures, table of epigenetic proteins, tables of potentially epigenome-interacting TCM medicinal. [file 816714.f1.pdf]

Epigenetics in Traditional Chinese Pharmacy: A Bioinformatic Study at Pharmacopoeia Scale

Hsin-Ying Hsieh [1], Pei-Hsun Chiu [1] & Sun-Chong Wang [1, 2, *]

[1] Institute of Systems Biology and Bioinformatics, National Central University, Chungli Taoyuan, 32001 Taiwan

[2] Epigenetics Laboratory, Centre for Addiction and Mental Health, Toronto Ontario M5T 1R8 Canada

* To whom correspondence should be addressed. E-mail: scwang@ncu.edu.tw

Key Words: *DNA methylation, histone modification, traditional Chinese medicine, DNMT, HDAC, HAT, HMT, MBD, Polycomb group protein, RNAi*

Online Supplementary Information

Data analysis

Overrepresentation analysis by the hypergeometric distribution: A TCM medicinal can possess a TCM nature Y or not. A collection of N medicinals contain m Y-medicinals and $N–m$ nonY-medicinals. The chance of getting k Y-medicinals from a sequence of n draws from the collection of N medicinals without replacement is given by the hypergeometric function. If the chance is lower than a cutoff, say, 0.05, and $k/n > m/N$, we say that Y-medicinals are overrepresented in the group of n medicinals. Y can be a taxonomy, TCM nature, flavour, meridian, or clinical function and so on. Note that since there are many natures (or flavours, and so on), the Bonferroni method is handy to adjust the p-values for multiple testing.

Probability of 198 epigenetic TCM formulas by chance: The 200 TCM formulas are made from 230 different medicinals, 115 of which are non-epigenetic. A formula is made up of 8 medicinals on average. In forming a formula by randomly drawing from the 230 medicinals, the chance of getting 8 non-epigenetic medicinals from 8 draws without replacement is $p = 0.075$ from the above hypergeometric function. The chance of forming an epigenetic formula where at least one medicinal among the 8 is epigenetic is therefore $q = 1 - p = 0.925$, which is high. However, the chance that this happens 198 times in 200 formula formulations is $C(200,198) \times q^{198} \times p^2 = 2.2 \times 10^{-5}$, where $C(200,198)$ is the binomial coefficient equal to $200 \times 199 / 2 / 1$.

Quantification of epigenetic role in TCM formulas: The aim was approached by an answer to how accurate are formulas' functions determined solely by the epigenome- and miRNA-interactivity of the medicinals in the formulas. This technically is a classifier validation problem in information science.

We adopted the *k*-nearest-neighbour algorithm (*k*NN) for classification and used the leave-one-out scheme for the evaluation of the classification accuracy [42]. In brief, a formula is picked up with its function masked. The formula is compared to each of the 199 formulas based on the epigenome- and miRNA-interactivity over the seven mechanisms (or the four TCM positions). The *k* formulas, called *k* nearest-neighbours, which are epigenetically most similar to the picked-up one are identified and the function of the picked-up formula is then determined by the majority of the functions of the *k* nearest-neighbour formulas. The predicted function is then compared to the function which was intentionally masked. This process is run iteratively over every formula. The success rate is then determined after the iteration. Note that *k* is a parameter in the *k*NN algorithm. The set of *k* nearest-neighbours may not be unique as, say, *k* + 5 formulas can have the same epigenetic similarity to the picked-up one. We varied *k* from 1, 3, 5, 7, …, up to 99, and repeated each value of *k* 100 times. The result in Fig. 9 represents the distribution of the accuracies from such repeated *k*-value optimizations.

Determination of a medicinal's TCM position: We determined *Monarch*, *Minister*, *Assistant* and *Guide* medicinals in a formula by the following algorithm: `the smallest integer not less than (i / (no. of medicinals / 4))`, where `i` is the order of the medicinal in the formula that composes more than four medicinals. For example, for a 5-medicinal formula, the result for `i` from 1 to 5 is 1, 2, 3, 4, 4, where 1's are *Monarch*, 2's are *Minister*, 3's are *Assistant* and 4's are *Guide*. For a 6-medicinal formula, it is 1, 2, 2, 3, 4, 4. For a 7-medicinal formula, it is 1, 2, 2, 3, 3, 4, 4. For an 8-medicinal formula, it is 1, 1, 2, 2, 3, 3, 4, 4. If a formula has only 2 (3) medicinals, it has only *Monarch* and *Minister* (and *Assistant*). With the algorithm, the average weights in grams were found to be 5.3, 4.5, 3.6, 2.9 for the *Monarch*, *Minister*, *Assistant* and *Guide* respectively, consistent with the general rule of TCM formulation that *Monarch* dosage is heaviest, followed by *Minister*, *Assistant* and *Guide*. Note that in the calculation of such quantities as weight, epigenome- and miRNA-

interactivity of a position, e.g. *Monarch*, the quantity was normalized by the total number of *Monarch*s in the formula.

Classification of new formulas by epigenome- and miRNA-interactivity: For the 13-medicinal TCM formula for SARS-like disease [40], the result for i from 1 to 13 is 1, 1, 1, 2, 2, 2, 3, 3, 3, 4, 4, 4, 4. The names and doses of the composing medicinals are listed in Table 2 of [40] available at http://ecam.oxfordjournals.org/cgi/content/full/5/3/355/T2. The epigenome- and miRNA-interactivity of the *Monarch* of the SARS-like formula can then be determined to be $(0\times16 + 6\times12 + 2\times12)/3$, where the 1st , 2nd and 3rd *Monarch* medicinals, having weights 16g, 12g and 12g, exploit respectively 0, 6 and 2 epigenetic mechanisms according to our list of epigenetic medicinals in Tables S3-S9. The epigenome- and miRNA-interactivity over the four positions of the formula was thus determined and compared to those of the 200 formulas using *k*NN. For $k > 11$, the algorithm invariantly returned *fire-purging*. For $k < 13$, the returned categories were *fire-purging*, *wind-expelling* and *gynaecologic*.

Online Supplementary Information Figures

Fig. S1

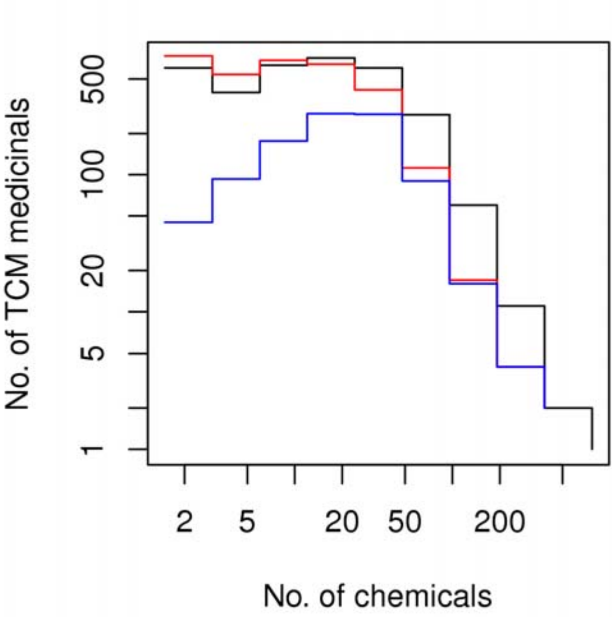

Fig. S1: Number of chemicals per TCM medicinal. 3,294 TCM medicinals have at least one chemical in their annotations in the integrated STDC/TCM-ID database. The median and mean numbers of chemicals per medicinal are 9 and 15. 65.5% of the chemicals come with CAS registry numbers, based on which we made the chemical-protein association. The distribution of the CAS-numbered chemicals is in red. 981 out of the 3,294 TCM medicinals are epigenome- and miRNA-interacting. The distribution of the numbers of chemicals per epigenetic TCM medicinal is shown in blue, the median and mean numbers being 13 and 18. Note of the double log scale of the plot.

Fig. S2

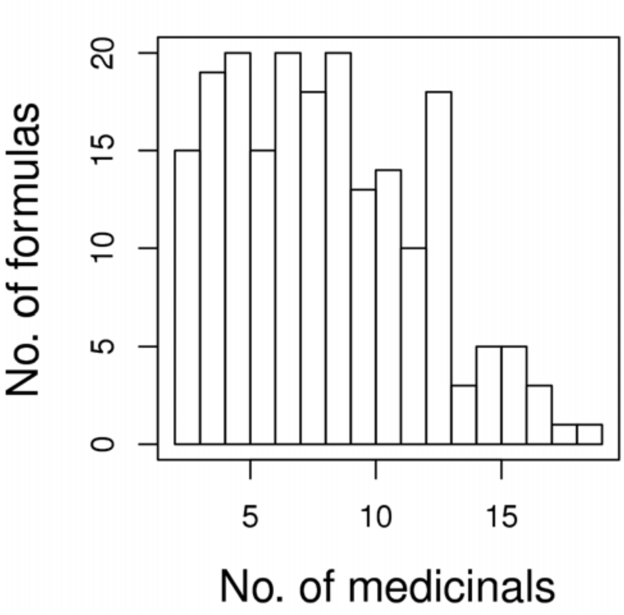

Fig. S2: Distribution of numbers of medicinals per formula. The minimum and maximum numbers of medicinals per formula are 2 and 19 respectively. The median number of medicinals per formula is 8.

Fig. S3

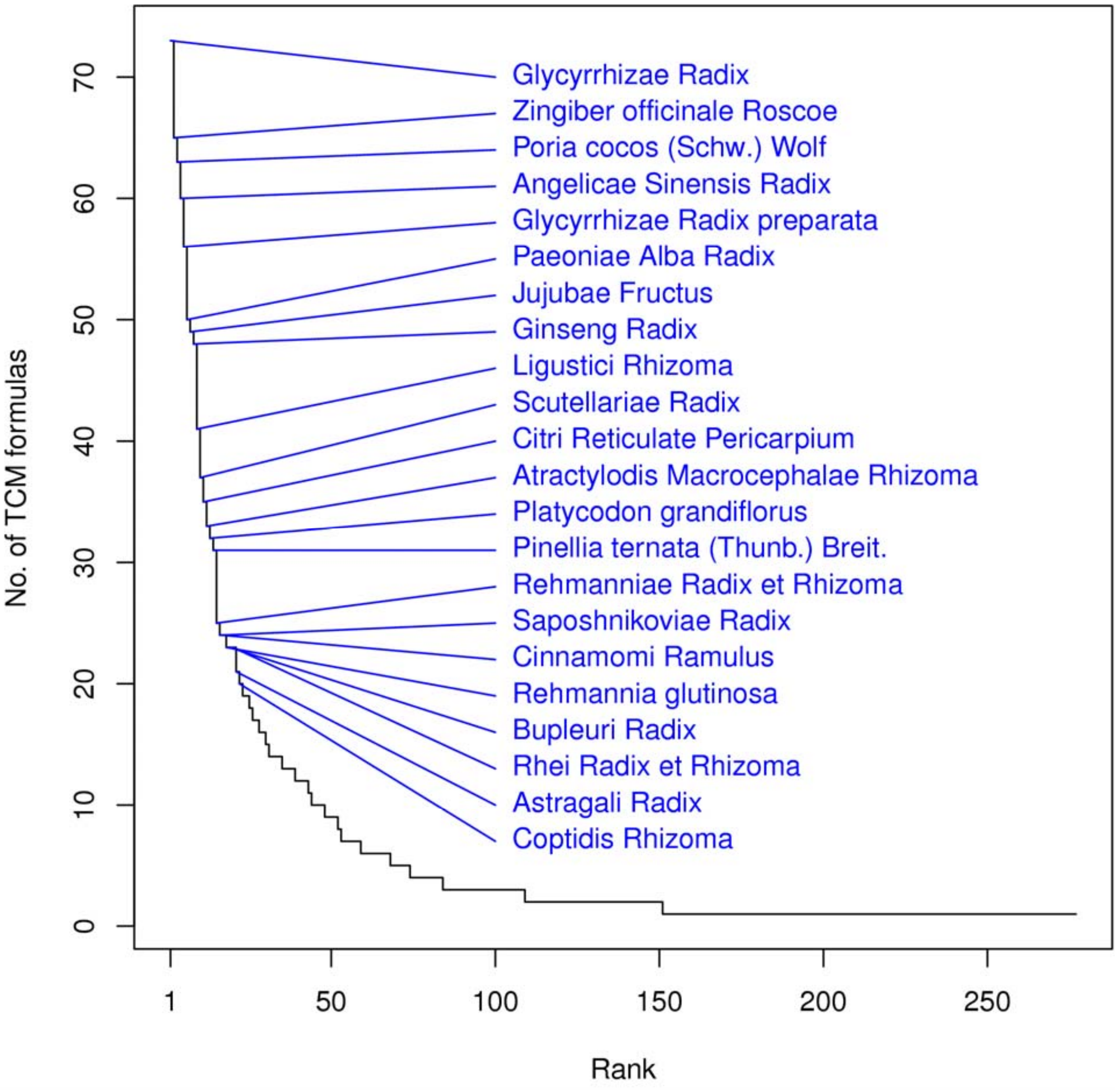

Fig. S3: Frequency distribution of the medicinals in the 200 TCM formulas. Medicinals are ranked according to their occurrence in the formulas. Also shown are the medicinals which appear in more than 20 formulas.

Fig. S4

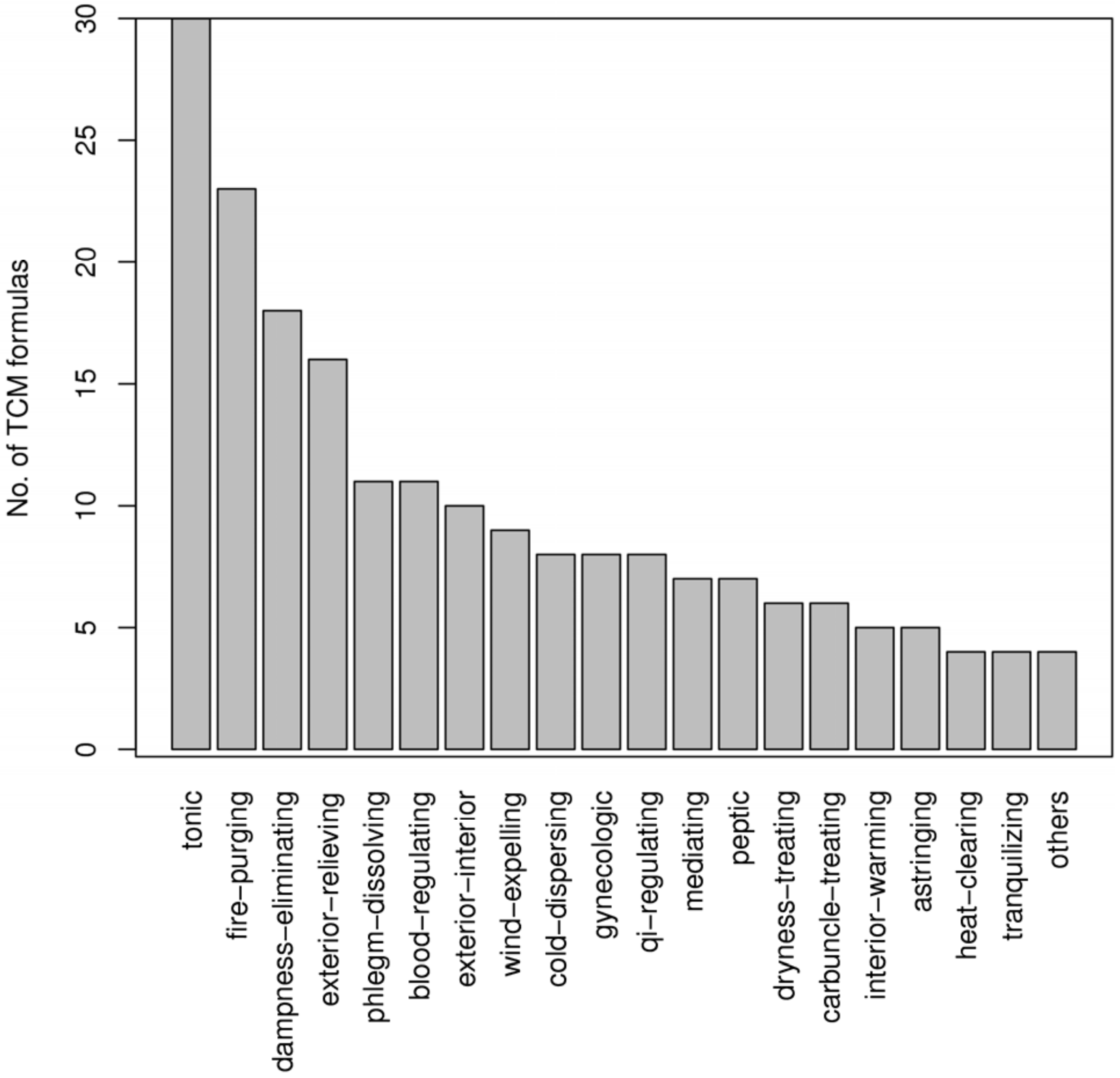

Fig. S4: Frequency distribution of the functional categories of the 200 TCM formulas. The function of a TCM formula is related to its therapeutic application. Twenty functions, including ‘others’, were known for the 200 formulas.

Fig. S5

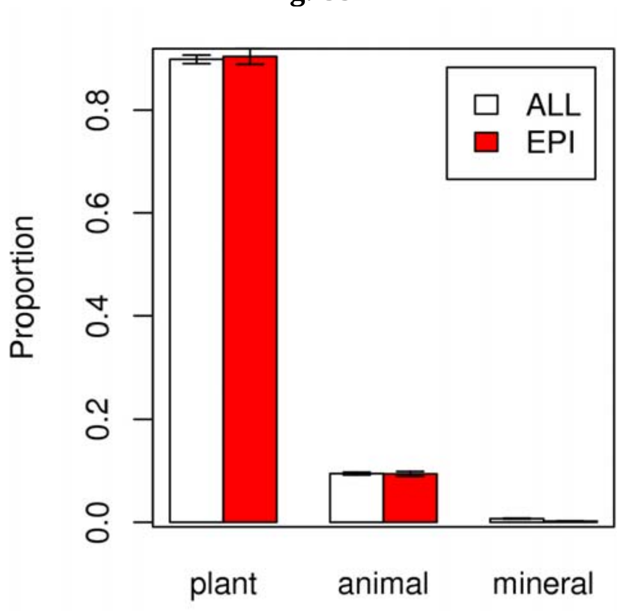

Fig. S5: Distribution of TCM medicinals' kingdoms. 3,284 TCM medicinals have kingdom annotation. Among them 980 are epigenetic and their proportions are shown in red. There are more plant epigenetic TCM medicinals than one would expect from the distribution of the 3,284 medicinals (p = 0.041).

Fig. S6

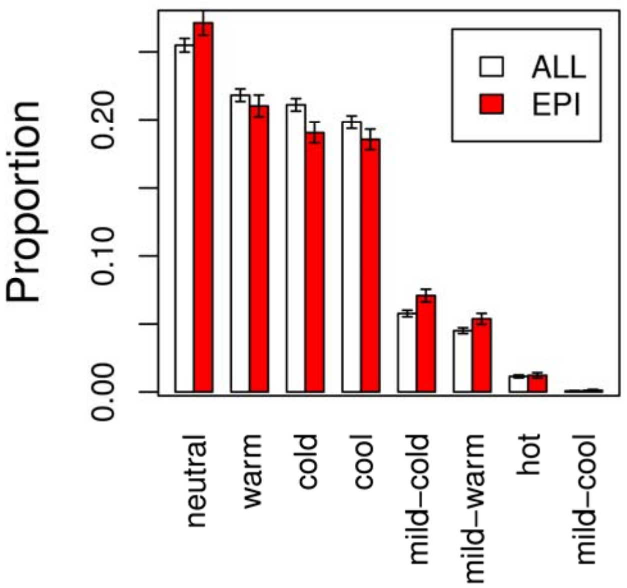

Fig. S6: Distribution of TCM natures. 2,530 TCM medicinals have TCM nature annotation. Among them 818 are epigenetic and their nature distribution is shown in red. There are more *mild-cold*, *neutral* and *mild-warm* (p = 0.011, 0.016 and 0.028 respectively) epigenetic TCM medicinals than one would expect from the nature distribution of the 2,530 medicinals.

Fig. S7

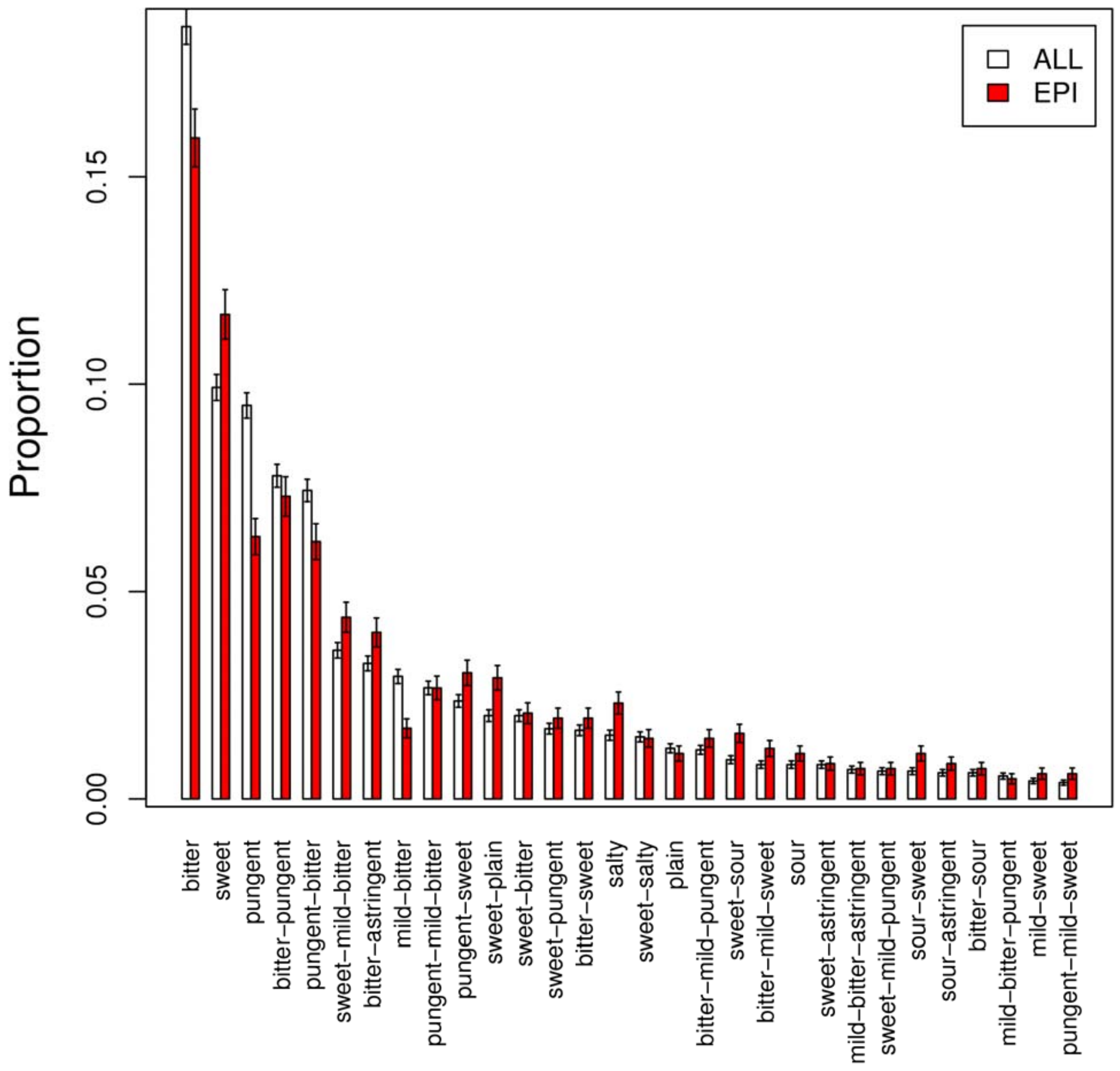

Fig. S7: Distribution of the top 30 TCM flavors. 2,540 TCM medicinals have flavor annotation. Among them 822 are epigenetic and their proportions are shown in red. There are more *sweet* (p = 0.0071) epigenetic TCM medicinals than one would expect from the flavor distribution of the 2,540 medicinals at a p-value cutoff of 0.01. Note that we treated *sweet-sour* and *sour-sweet*, as well as other reverse-order flavor combinations, as different flavors.

Fig. S8

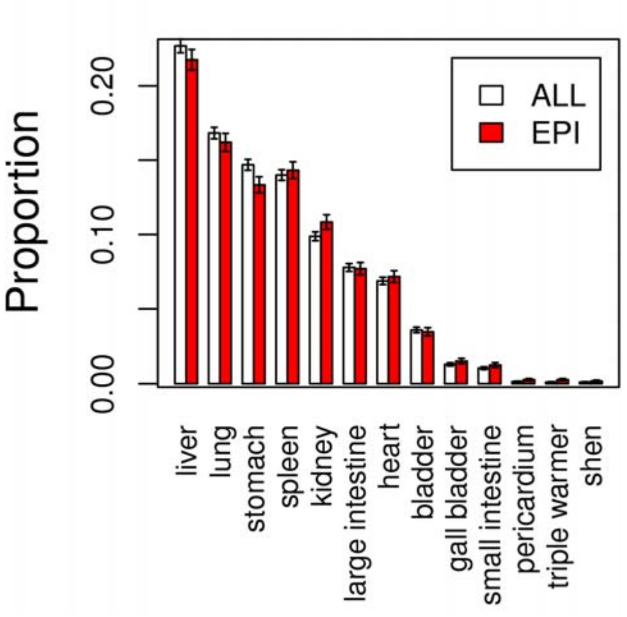

Fig. S8: Distribution of TCM meridians. 1,155 TCM medicinals have meridian annotation. Among them 452 are epigenetic and their meridian proportions are shown in red. Note that unlike natures and flavors, a TCM medicinal can possess more than one meridians. There are more *kidney* and *spleen* (*p* = 0.020 and 0.041 respectively) epigenetic TCM medicinals than one would expect from the meridian distribution of the 1,155 medicinals.

Fig. S9

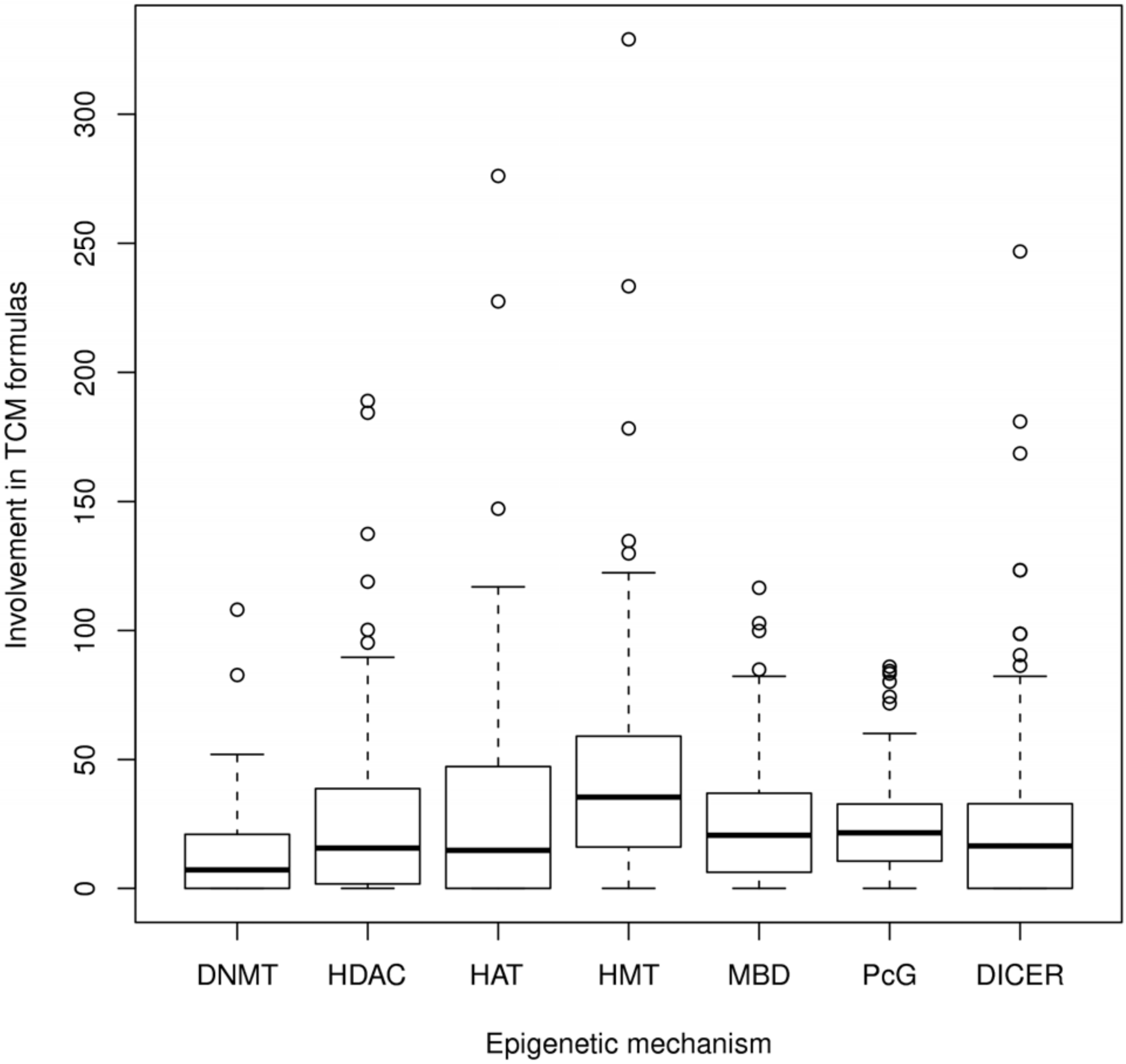

Fig. S9: Role of specific mechanisms in the 198 epigenetic TCM formulas. The 'involvement' of the *y*-axis measures the accumulated evidence score multiplied by the grams of the medicinals in the formula.

Fig. S10

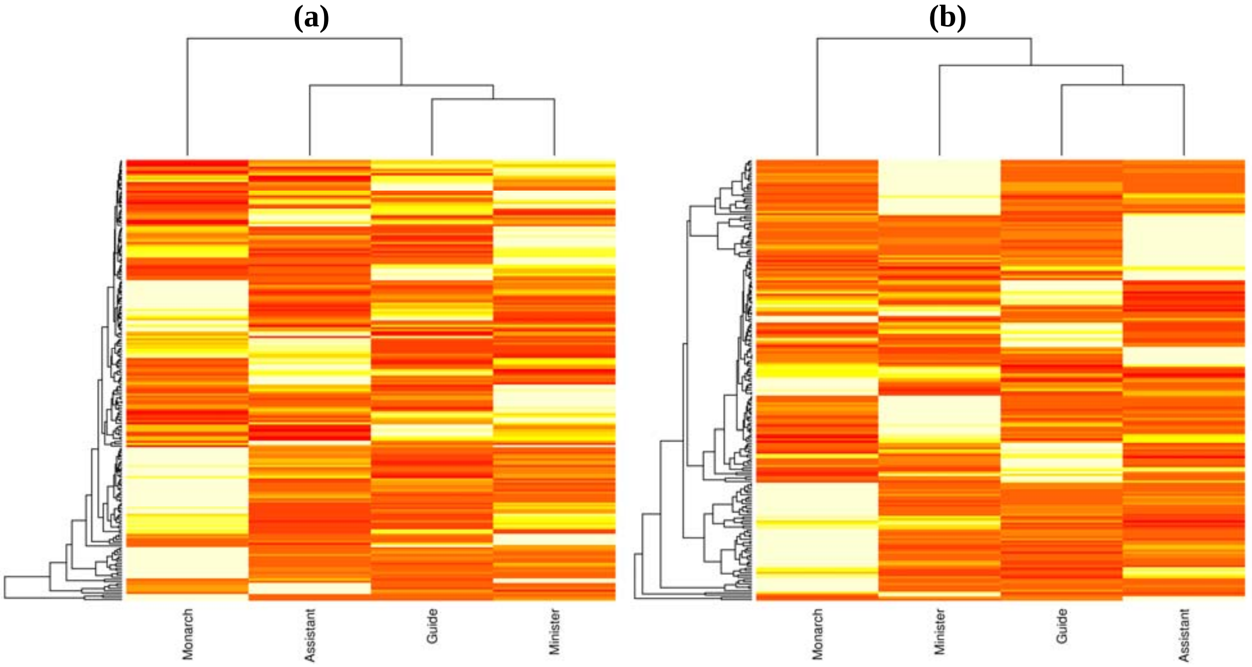

Fig. S10: Epigenome- and miRNA-interactivity of the 198 epigenetic TCM formulas over TCM positions. Rows represent TCM formulas and columns the four TCM positions. The lighter the shades the stronger the epigenome- and miRNA-interactivity of the medicinals in the position. Orders of both rows and columns are arranged by the unsupervised hierarchical clustering algorithm. (a) considers no score while (b) considers no gram.

Fig. S11

(a) **(b)**

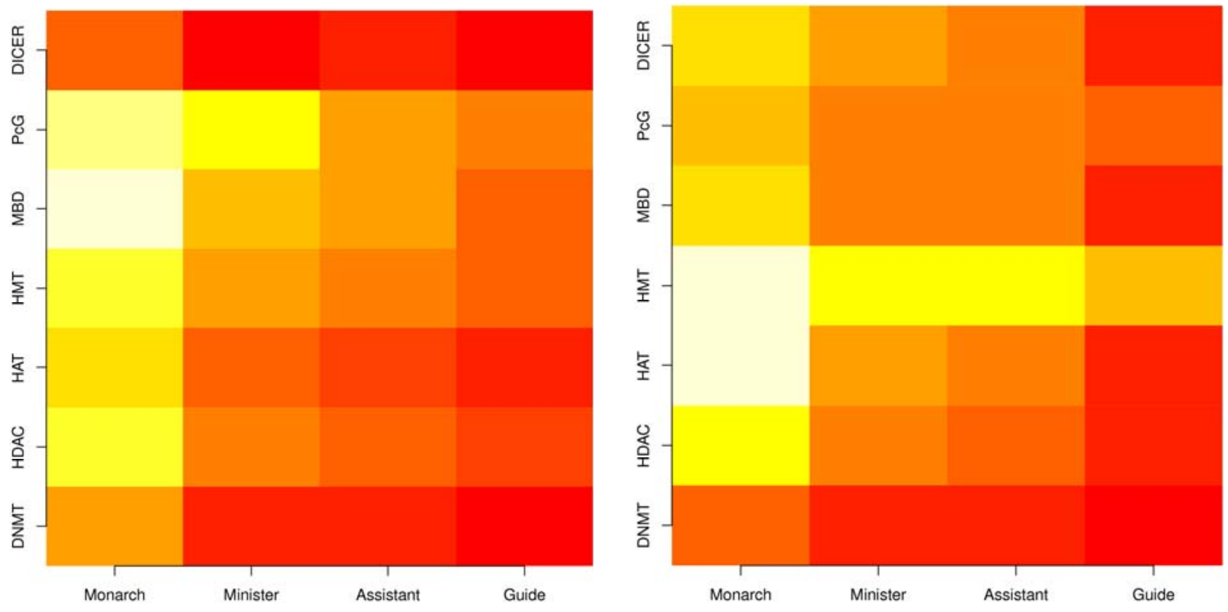

Fig. S11: Pattern of utilization of epigenetic mechanisms over TCM positions. Medicinals in a formula can be differentiated by their positions in the formula into *Monarch*, *Minister*, *Assistant* and *Guide*. The mechanism-specific interactivity, without regard to the number of interacting chemicals in the medicinal and degree of evidence of the interactions, is shown in (a). The mechanism-specific interactivity, without regard to the doses of the medicinals, is shown in (b).The lighter the shades, the larger the interactivity.

Table S1 Epigenetics-related proteins

species_ncbi_taxon_id	protein_id	alias	source
9606	ENSP00000352516	DNMT	Ensembl_HUGO_Previous_Symbols Ensembl_Uniprot/SWISSPROT_GN
9606	ENSP00000352516	DNMT1	Ensembl_Uniprot/SWISSPROT_GN HUGO
9606	ENSP00000312697	DNMT1-associated protein 1	Ensembl_Uniprot/SWISSPROT_DE
9606	ENSP00000352516	DNMT1_HUMAN	Uniprot/SWISSPROT
9606	ENSP00000346652	DNMT2	Ensembl_Uniprot/SWISSPROT_GN HUGO
9606	ENSP00000346652	DNMT2_HUMAN	Uniprot/SWISSPROT
9606	ENSP00000264709	DNMT3A	Ensembl_Uniprot/SWISSPROT_GN HUGO
9606	ENSP00000328547	DNMT3B	Ensembl_Uniprot/SWISSPROT_GN HUGO
9606	ENSP00000270172	DNMT3L	Ensembl_Uniprot/SWISSPROT_GN HUGO
9606	ENSP00000312697	DNMTAP1	Ensembl_HUGO_Aliases
9606	ENSP00000352516	Dnmt1	Ensembl_Uniprot/SWISSPROT_DE
9606	ENSP00000346652	Dnmt2	Ensembl_Uniprot/SWISSPROT_DE
9606	ENSP00000264709	Dnmt3a	Ensembl_Uniprot/SWISSPROT_DE
9606	ENSP00000328547	Dnmt3b	Ensembl_Uniprot/SWISSPROT_DE
9606	ENSP00000338617	Histone deacetylase complex subunit Sin3a	Ensembl_Uniprot/SWISSPROT_DE
9606	ENSP00000271095	histone deacetylase 1	Ensembl_HUGO_Approved_Name
9606	ENSP00000216271	histone deacetylase 10	Ensembl_HUGO_Approved_Name
9606	ENSP00000295757	histone deacetylase 11	Ensembl_HUGO_Approved_Name
9606	ENSP00000347104	histone deacetylase 2	Ensembl_HUGO_Approved_Name
9606	ENSP00000302967	histone deacetylase 3	Ensembl_HUGO_Approved_Name
9606	ENSP00000264606	histone deacetylase 4	Ensembl_HUGO_Approved_Name
9606	ENSP00000225983	histone deacetylase 5	Ensembl_HUGO_Approved_Name
9606	ENSP00000334061	histone deacetylase 6	Ensembl_HUGO_Approved_Name
9606	ENSP00000080059	histone deacetylase 7A	Ensembl_HUGO_Approved_Name
9606	ENSP00000354431	histone deacetylase 8	Ensembl_HUGO_Approved_Name
9606	ENSP00000262069	histone deacetylase 9	Ensembl_HUGO_Approved_Name
9606	ENSP00000225916	Histone acetyltransferase GCN5	Ensembl_Uniprot/SWISSPROT_DE
9606	ENSP00000287239	Histone acetyltransferase MORF	Ensembl_Uniprot/SWISSPROT_DE
9606	ENSP00000287239	Histone acetyltransferase MOZ2	Ensembl_Uniprot/SWISSPROT_DE
9606	ENSP00000259021	Histone acetyltransferase	Ensembl_Uniprot/SWISSPROT_DE

		binding to ORC1	
9606	ENSP00000331679	Histone acetyltransferase type B subunit 2	Ensembl_Uniprot/SWISSPROT_DE
9606	ENSP00000219797	MYST histone acetyltransferase 1	Ensembl_HUGO_Approved_Name
9606	ENSP00000259021	MYST histone acetyltransferase 2	Ensembl_HUGO_Approved_Name
9606	ENSP00000265713	MYST histone acetyltransferase 3	Ensembl_HUGO_Approved_Name
9606	ENSP00000287239	MYST histone acetyltransferase 4	Ensembl_HUGO_Approved_Name
9606	ENSP00000264108	histone acetyltransferase 1	Ensembl_HUGO_Approved_Name
9606	ENSP00000221482	DOT1-like histone H3 methyltransferase	Ensembl_HUGO_Approved_Name
9606	ENSP00000298728	Euchromatic histone-lysine N-methyltransferase 1	Ensembl_Uniprot/SWISSPROT_DE
9606	ENSP00000259865	Euchromatic histone-lysine N-methyltransferase 2	Ensembl_Uniprot/SWISSPROT_DE
9606	ENSP00000221482	Histone H3 K79 methyltransferase	Ensembl_Uniprot/SWISSPROT_DE
9606	ENSP00000258672	Histone H3 K9 methyltransferase	Ensembl_Uniprot/SWISSPROT_DE
9606	ENSP00000332183	Histone H3 K9 methyltransferase 1	Ensembl_Uniprot/SWISSPROT_DE
9606	ENSP00000346997	Histone H3 K9 methyltransferase 2	Ensembl_Uniprot/SWISSPROT_DE
9606	ENSP00000259865	Histone H3 K9 methyltransferase 3	Ensembl_Uniprot/SWISSPROT_DE
9606	ENSP00000271640	Histone H3 K9 methyltransferase 4	Ensembl_Uniprot/SWISSPROT_DE
9606	ENSP00000298728	Histone H3 K9 methyltransferase 5	Ensembl_Uniprot/SWISSPROT_DE
9606	ENSP00000262189	Histone- lysine N-methyltransferase, H3 lysine 4 specific MLL3	Ensembl_Uniprot/SWISSPROT_DE
9606	ENSP00000298728	euchromatic histone-lysine N-methyltransferase 1	Ensembl_HUGO_Approved_Name
9606	ENSP00000318085	Methyl CpG-binding protein 2- interacting zinc finger protein	Ensembl_Uniprot/SWISSPROT_DE
9606	ENSP00000269468	Methyl CpG-binding protein MBD1	Ensembl_Uniprot/SWISSPROT_DE

9606	ENSP00000256429	Methyl CpG-binding protein MBD2	Ensembl_Uniprot/SWISSPROT_DE
9606	ENSP00000156825	Methyl CpG-binding protein MBD3	Ensembl_Uniprot/SWISSPROT_DE
9606	ENSP00000249910	Methyl CpG-binding protein MBD4	Ensembl_Uniprot/SWISSPROT_DE
9606	ENSP00000347896	Methyl CpG-binding protein MBD6	Ensembl_Uniprot/SWISSPROT_DE
9606	ENSP00000269468	Protein containing methyl CpG-binding domain 1	Ensembl_Uniprot/SWISSPROT_DE
9606	ENSP00000301948	methyl CpG binding protein 2	Ensembl_HUGO_Approved_Name
9606	ENSP00000269468	methyl-CpG binding domain protein 1	Ensembl_HUGO_Approved_Name
9606	ENSP00000256429	methyl-CpG binding domain protein 2	Ensembl_HUGO_Approved_Name
9606	ENSP00000156825	methyl-CpG binding domain protein 3	Ensembl_HUGO_Approved_Name
9606	ENSP00000304198	methyl-CpG binding domain protein 3-like 1	Ensembl_HUGO_Approved_Name
9606	ENSP00000351254	methyl-CpG binding domain protein 3-like 2	Ensembl_HUGO_Approved_Name
9606	ENSP00000249910	methyl-CpG binding domain protein 4	Ensembl_HUGO_Approved_Name
9606	ENSP00000347896	methyl-CpG binding domain protein 6	Ensembl_HUGO_Approved_Name
9606	ENSP00000269397	Polycomb 2 homolog	Ensembl_Uniprot/SWISSPROT_DE
9606	ENSP00000269385	Polycomb 3 homolog	Ensembl_Uniprot/SWISSPROT_DE
9606	ENSP00000302780	Polycomb complex protein BMI 1	Ensembl_Uniprot/SWISSPROT_DE
9606	ENSP00000263062	enhancer of polycomb homolog 1	Ensembl_HUGO_Approved_Name
9606	ENSP00000258484	enhancer of polycomb homolog 2	Ensembl_HUGO_Approved_Name
9606	ENSP00000233630	polycomb group ring finger 1	Ensembl_HUGO_Approved_Name
9606	ENSP00000350157	polycomb group ring finger 5	Ensembl_HUGO_Approved_Name
9606	ENSP00000286910	polycomb group ring finger 6	Ensembl_HUGO_Approved_Name
9606	ENSP00000343745	DICER	Ensembl_Uniprot/SWISSPROT_GN
9606	ENSP00000343745	DICER1	Ensembl_Uniprot/SWISSPROT_GN HUGO
9606	ENSP00000343745	DICER_HUMAN	Uniprot/SWISSPROT
9606	ENSP00000343745	Dicer	Ensembl_HUGO_Aliases

9606	ENSP00000343745 Dicer1	Ensembl_HUGO_Approved_Name
9606	ENSP00000343745 Dicer1 Dcr-1 homolog	Ensembl_HUGO_Approved_Name
9606	ENSP00000339845 Drosha	Ensembl_Uniprot/SWISSPROT_DE

Table S2 Pair-wise epigenetic distance between positions

Gram & score			
	Monarch	*Minister*	*Assistant*
Minister	1754		
Assistant	2028	1100	
Guide	1873	802	908
Gram & no score			
	Monarch	*Minister*	*Assistant*
Minister	307		
Assistant	337	201	
Guide	319	161	174
Score & no gram			
	Monarch	*Minister*	*Assistant*
Minister	250		
Assistant	258	199	
Guide	232	165	155

Table S3 DNMT-interacting TCM medicinals

DNMT
Melothria heterophylla (Lour.) Cogn., root, 茅瓜
Dioscorea opposita Thunb., rhizome of Common Yam, 山药
Bupleurum komarovianum Lincz., root of Siberian Thorowax, 柴胡
Cryptotympana pustulata Fabricius, Cicada slough, 蝉蜕
Panax notoginseng (Burk.) F.H.Chen, Sanchi, 三七
Angelica sinensis (Oliv) Diels., root of Chinese Angelica, 当归
Angelica pubescens f. biserrata Shan et Yuan, root of Doubleteeth pubescent Angelica, 独活
Arisaema elephas S.Buchet, tuber of korean Jackinthepulpit, 天南星
Paris polyphylla var.chinensis, petiolata Parus Rhizome, 蚤休
Phytolacca acinosa Roxb., root of Indian Pokeweed, 商陆
Stellera chamaejasme L., root, 狼毒
Changium smyrnioides Wolff, root of Medicinal Changium, 明党参
Rehmannia glutinosa (Gaertn.) Libosch., fresh rhizome of Adhesive Rehmannia, 鲜地黄
Coriolus versicolor (Lr.) Quel, Polystictus versicolor (L.) Fr., 云芝
Rheum palmatum L., root and rhizome of Sorrel Rhubarb, 大黄
Melia azedarach L., bark of Szechwan chinaberry, 苦楝皮
Acanthopanax giraldii Harms, bark of Girald Acanthopanax, 红毛五加皮
Pyrus pashia Buch.-Ham.ex D. Don, bark, 川梨茎皮
Oxytropis glabra DC., all-grass of Glabrous Crazyweed, 醉马草
Vladimiria souliei (Franch.)Ling, root of Common Aucklandia, 木香
Pheretima aspergillum (E. Perrier) dried body, 地龙
Ligustrum lucidum Ait., fruit of Glossy privet, 女贞子
Lycopersicon esculentum Mill., Tomato, 番茄
Lappula myosotis Moench, Common Carpesium Fruit, 赖毛子
Spinacia oleracea L., all-grass of Spinach, 菠菜
Vicia sativa L., all-grass of Common vetch, 大巢菜
Astragalus membranaceus (Fisch.) Bge. Var. Monghol, Radix Astragali, 黄芪
Bambusa textilis Mc-Clure, CONCRETIO SILICEA BAMBUSAE, 竹黄
Senecio chrysanthemoides DC., all-grass of Chrysanthemum-like Groundsel, 土三七
Citrullus lanatus (Thurb.) Mansfeld, Pulp of a Watermelon, 西瓜
Chinemys reevesii (Gray), Carapax Et Plastrum Testudinis, 龟甲
Euphoria longan (Lour). Steud., Longan aril, 龙眼肉
Ganoderma lucidum, Lingzhi mushroom, 灵芝

Asterias rollestoni, sea star, 海盘车
Plantago asiatica L., seed of Depressed plantain, 车前子
Chaenomeles japonica (Thunb.) Lindl. ex Spach, Japanese papaya, 和木瓜
Lycium dasystemum pojark., fruit of Hairystamen Wolfberry, 枸杞子
Lepisma saccharina L., silverfish, 衣鱼
Rana nigromaculata Hallowell, Pond green frog, 青蛙
Glyptostrobus pensilis (Staunt.) Koch [Thuja pensilis Staunt.], leaf, 水松
Luffa acutangula Roxb., Seed of Towel Gourd, 丝瓜子
Pleurotus ostreatus (Jacg:Fr.)Kummer, mushroom, 侧耳
Hvdrangea paniculata Sieb., root or leaf, 水亚木
Panax ginseng C.A.Mey., Ginseng root, 人参
Saccharum sinensis Roxb., Sugarcane, 甘蔗
Tussilago farfara L., Common Coltsfoot Flower, 款冬花
Cucurbita moschata (Duch.) Poiret, furit of Cushaw, 南瓜
Cordyceps militaris, fungus that parasitises and kills moth pupae, 蛹草
Cordyceps sinensis (Bark.). Sacc., Chinese Caterpillar fungus, 冬虫夏草
Pisum sativum L., Pea, 豌豆
Amomum subulatum Roxb., fruit of Fragrant Amomum, 香豆蔻
Raphanus sativus L., Radish root, 莱菔
Hedysarum polybotrys Hand.-Mazz., root of Manyinflorescenced Sweetvetch., 红芪
Astragalus sinicus L., all-grass of Chinese Milkvetch., 红花菜
Hordeum Vulgare L., Malt, 麦芽
Misgurnus anguillicaudatus (Cantor), Oriental weatherfish, 泥鳅
Oryza sativa L., unhusked rice sprout, 谷芽
Gallus gallus domesticus Brisson, Chicken brains, 鸡脑
Imperata Cylindrica Beauv. var. major (Nees) C.E.Hubb., Leaf of LaLang Grass, 茅草叶
Isatis tinctoria L., root of Indigowoad, 板蓝根
Levisticum officinale Koch, root of Garden Lovage, 欧当归
Dryopteris filix-mas (L.) Schott, rhizome, 欧绵马
Morus alba L., Mulberry leaf, 桑叶
Castanea mollissima Bl., leaf of Hairy Chestnut, 栗叶
Castanea mollissima Bl., bark of Hairy chesstnut, 栗树皮
Castanea mollissima Bl., root of Hairy chestnut, 栗树根
Herba Zosterae Marinae, all grass, 大叶藻
Ranarugosa (schlegel)., toad, 粗皮蛙

Agama hmalagana, Tibetan Gecko, 藏蛤蚧
Allium fistulosum L., Fistular onion leaf, 葱叶
Trachycarpus fortunei H.Wendl., flower of Fortune Windmillpalm, 棕榈花

Table S4 HDAC-interacting TCM medicinals

HDAC

Salsola ruthenica Iljin, all-grass of Russianthistle, 刺沙蓬

Harengula zunasi (Bleeker), Pilchard, 青鳞鱼

Securinega suffruticosa (Pall.) Rehd., twig and leaf of suffrutesent Securinega, 一叶萩

Melothria heterophylla (Lour.) Cogn., root, 茅瓜

Artemisia capillaris Thunb., seedling of Capillary Wormwood, 茵陈蒿

Carica papaya L., Papaya, 番木瓜

Imperata cylindrica Beauv.var.major (Nees) C.E.Hubb., rhizome of Lalang Grass, 白茅根

Cistanche salsa (C.A.Mey) Benth.et Hook.f., Saline Cistanche, 肉苁蓉

Capsella brusa-pastoris (L.) Medic., Herba Capsellae, 荠菜

Dioscorea opposita Thunb., rhizome of Common Yam, 山药

Alocasia cucullata (Lour.) Schott, rhizome of Hoodshaped Alocasia, 卜芥

Cynanchum thesioides (Freyn) K.Schum., all-grass of Bastard. toadflax-like swallowwort, 地梢瓜

Salvia miltiorrhiza Bunge, root of Dan-shen, 丹参

Rubus corchorifolius L. F., root and leaf, 山莓

Bupleurum komarovianum Lincz., root of Siberian Thorowax, 柴胡

Crataegus pinnatifida Bunge, fruit of Chinese Hawthorn, 山楂

Pyrus pyrifolia (Burm.f.) Nakai, fruit of Bretschneider pear, 梨

Averrhoa carambola L., fruit of Common Averrhoa, 阳桃

Allium victorialis, wild onion, 茖葱

Panax notoginseng (Burk.) F.H.Chen, Sanchi, 三七

Angelica sinensis (Oliv) Diels., root of Chinese Angelica, 当归

Buthus martensii Karsch., Scorpion, 全蝎

Fortunella crassifolia Swingle, fruit of Oval Kumquat, 金橘

Punica granatum L., Pomegranate rind, 石榴皮

Sarcandra glabra (Thunb.) Nakai, all-grass of Glabrous sarcandra, 肿节风

Pteridium aquilinum (L.) Kuhn var.latiusculum (Desv.) Underw, all-grass of Eastern Bracken Fern, 蕨

Rosa laevigata Michx., Cherokee Rose Fruit, 金樱子

Phlomis umbrosa Turcz., root or all-grass of Shady Jerusalemsage, 糙苏

Stemona japonica (Bl).Miq., root of Japanese Stemona, 百部

Quisqualis indica L., fruit of Rangooncreeper, 使君子

Osbeckia chinensis L., all-grass of Chinese osbeckia, 天香炉

Phyllanthus urinaria L., whole plant, 叶下珠

Arisaema elephas S.Buchet, tuber of korean Jackinthepulpit, 天南星
Cornus officinalis Sieb. et Zucc., fruit of Asiatic Cornelian cherry, 山茱萸
Solanum tuberosum L., potato, 马铃薯
Anemarrhena asphodeloides Bunge, rhizome of Common Anemarrhena, 知母
Clausena excavata Burm.f., bark of Hollowed Wampee, 山黄皮
Stellera chamaejasme L., root, 狼毒
Daucus carota L., all-grass of Wild Carrot, 鹤虱风
Viburnum odoratssimum Ker-Gawl., root of Sweet Viburnum, 旱禾树
Boehmeria tricuspis (Hance) Makino, root, 山麻根
Vitis vinifera L., fruit of European Grape, 葡萄
Crataegus pinnatifida Beg. var. major N.E.Br/C. Cuneata sieb. et Zucc., Hawthorn seed, 山楂核
Ostericum grosserratrm (Maxim.) Kitag., root, 山水芹菜
Flemingia philippinesis Merr.et Rolfe, root of philippine Flemingia, 千斤拔
Murraya exotica L., Leaf and twig of Common Jasminorange, 九里香
Lespedeza cuneata (Dum.Cours.) G.Don, all-grass of Cuneate Lespedeza, 夜关门
Coriolus versicolor (Lr.) Quel, Polystictus versicolor (L.) Fr., 云芝
Eupatorium fortunei Turcz., all-grass of Fortune Eupatorium, 佩兰
Achillea alpina L., all-grass of Alpine Yarrow, 蓍草
Rheum palmatum L., root and rhizome of Sorrel Rhubarb, 大黄
Melia azedarach L., bark of Szechwan chinaberry, 苦楝皮
Citrus medica L. var,sarcodactylis (Noot) Swingle., fruit of Fleshfingered Citron, 佛手柑
Pyrus pashia Buch.-Ham.ex D. Don, bark, 川梨茎皮
Thalictrum baicalense Turcz., root and rhizome of Baikal Meadowrue, 马尾连
Arachis hypogaea L., Peanut, 落花生
Citrus sinensis (L.) Osbeck, fruit of Sweet Orange, 甜橙
Lysimachia foenum-graecum Hance, whole plant, 灵香草
Musa sapientum L., Banana, 香蕉
Ligustrum lucidum Ait., fruit of Glossy privet, 女贞子
Ardisia pusilla A.DC., whole plant, 小青
Foeniculum vulgare Mill., Fennel seed, 小茴香
Semen Dolichoris Album, White Hyacinth Bean, 白扁豆
Leibnitzia anandria (L.) Nakai, all-grass of Common Leibnitzia, 大丁草
Oenanthe javanica (Bl.) DC., all-grass of Javan Waterdropwort, 水芹
Galium aparine L. var. tenerum (Gren. et Godr.) Reichb., whole plant, 八仙草
Capsicum rutescens L., Hot pepper, 辣椒

Polemonium laxiflorum Kitam., root and rhizome, 花荵
Lycopersicon esculentum Mill., Tomato, 番茄
Duchesnea indica (Andr.) Focke., all-grass of Indian Mockstrawberry, 蛇莓
Schizonepeta tenuifolia Brig., all-grass of Fineleaf Schizonepeta, 荆芥
Sesamum indicum L., seed, 黑芝麻
Typha angustifolia L., pollen of Longbract Cattail., 蒲黄
Allium macrostemon Bunge, bulb of Longstamen Onion, 薤白
Chlorella pyrenoidosa Chick., algae, 小球藻
Polygonum amphibium L., all-grass of Amphibious Knotweed, 两栖蓼
Anemone altaica Fisch. Ex C.A.Mey., rhizome of Irkutsk Anemone, 九节菖蒲
Rubus lambertianus Ser. ex DC., root and leaf, 高梁泡
Polypodium vulgare L； Polypodium virginianum L., rhizome, 多足蕨
Liticauda semifasciata (Reinhardt), Erabu-unagi, 蛇婆
Desmodium gangeticum (L.) DC., stem and leaf of Hookedhairypod Tickclover, 红母鸡草
Viola philippica Cav.ssp. munda V.Beck., all-grass of Neat Philippine Violet, 紫花地丁
Lobelia chnensis Lour., all-grass of Chinese Lobelia, 半边莲
Lappula myosotis Moench, Common Carpesium Fruit, 赖毛子
Sparganium stoloniferum Buch.-Ham., rhizome of Common Burreed, 三棱
Armillariella mellea (Vahl.ex Fr.) Karst., fruiting body, 蜜环菌
Gnetum parvifolium (Warb.) C.Y.Cheng, Stem of Smallleaf Jointfir, 买麻藤
Oxya chinensis Thunb., locust, 蚱蜢
Blatta orientalis L., Oriental Cockroach, 蟑螂
Spinacia oleracea L., all-grass of Spinach, 菠菜
Osmunda japonice Thunb., Japanese Flowering Fern Rhizome, 紫萁贯众
Portulaca oleracea L., all-grass of Purslane, 马齿苋
Euphorbia helioscopia L., all-grass of Sun Euphorbia, 泽漆
Thalictrum alpinum L., root and rhizome of Alpine Meadowrue, 高山唐松草
Astragalus membranaceus (Fisch.) Bge. Var. Monghol, Radix Astragali, 黄芪
Hemidactylus, Gommon House Gecko, 蜥虎
Gastrodia elata Bl., tuber of Tall Gastrodia, 天麻
Arisaema fargesii Buchet, tuber of Farges Jackinthepulpit, 螃蟹七
Saxifraga stolonifera (L.) Meerb., all-grass of Creeping Rockfoil, 虎耳草
Malus pumila Mill., Apple, 苹果
Senecio chrysanthemoides DC., all-grass of Chrysanthemum-like Groundsel, 土三七
Citrullus lanatus (Thurb.) Mansfeld, Pulp of a Watermelon, 西瓜

Equisetum hiemale L., horsetail, 木贼
Sarcostemma acidam (Roxb) Voigt, whole plant, 无叶藤
Ficus carica L., Fig, 无花果
Ziziphus jujuba Mill, Chinese date, 大枣
Sapindus mukorossi Gaertn., seed of Chinese soapberry, 无患子
Phallus impudicus L. ex. Pers., stem, 白鬼笔
Eriocheir sinensis H.Milne-Edwards, Carb, 蟹
Lepidium apetalum Willd., seed, 葶苈子
Phaseolus vulgaris, bean, 菜豆
Chaenomeles speciosa (Sweet) Nakai, fruit of Common Floweringquince, 木瓜
Xanthoceras sorbifolia Bunge, wood or twig and leaf of Shinyleaf Yellowhorn., 文冠果
Ganoderma lucidum, Lingzhi mushroom, 灵芝
Medicago sativa L., all grass, 苜蓿
Aegie marmelos (L.) Correa, fruit, 硬皮橘
Gossampinus malabarica (DC.) Merr., flower of Common Bombax, 木棉花
Gossypium herbaceum L., seed of Levant Cotton, 棉花子
Catharanthus roseus (L.) G.Don, all-grass of Madagascar Periwinkle, 长春花
Asterias rollestoni, sea star, 海盘车
Pseudostellaria heterophylla (Miq.) Pax ex Pax et , Radix Pseudostellariae, 太子参
Tetraplodon mnioides (Hedw.) B.S.G., moss, 并齿藓
Penaeus orientalis Kish., lobster, 对虾
Plantago asiatica L., seed of Depressed plantain, 车前子
Macrocystis pyrifera (L.) Ag., algae, 巨藻
Urtica macrorrhiza Hand.-Mazz., whole plant, 青活麻
Spirulina, algae, 螺旋藻
Callicarpa macrophylla vahl, root or leaf of Bigleaf Beautyberry, 大叶紫珠
Mustelus manazo Bleeder, shark, 鲨鱼肉
Lycium dasystemum pojark., fruit of Hairystamen Wolfberry, 枸杞子
Anguilla japonica Temminck et Schlegel, Japanese eel, 鳗鲡鱼
Bubalus bubalis L., Ox blood, 牛血
Bubalus bubalis L., milk, 牛乳
Vaccinium bracteatum Thunb., fruit of Oriental Blueberry, 南烛子
Cynanchum paniculatum (Bge.) Kitag., rhizome, 牛心朴
Annona reticulata L., fruit, 牛心果
Xanthium sibircium Patr. ex Widd., fruit of Siberian Cocklebur, 苍耳子

Capparis zeylanica L., root-bark of Ceylon Caper, 槛藤子
Rhaponticum uniflorum (L.) DC., root, 漏芦
Typhonium giganteum Engl., rhizome, 白附子
Lawsonia inermis L., Henna leaf, 指甲花叶
Eucalyptus citriodora, leaf, 柠檬桉叶
Elsholtzia criostachya Benth., all grass, 黄花香薷
Rana nigromaculata Hallowell, Pond green frog, 青蛙
Glyptostrobus pensilis, bark and leaf, 水松
Thesium chinensis Turcz., all-grass of Chinese Bastardtoadflax., 百蕊草
Nicotiana tabacum L., leaf of Common Tobacco, 烟草
Allium cepa L., bulb of Common Onion, 洋葱
Ginkgo biloba L., seed of Maidenhairtree, 白果
Drymaria cordata (L.) Willd., all-grass of West Indian chickweed, 荷莲豆菜
Zebrina pendula Schnizl., all-grass of Wanderingjew Zebrina, 吊竹梅
Litchi chinensis Sonn., Lychee Nut, 荔枝
Glycine max.(L.) Merr., black seed of Soybean., 黑大豆
Prunus mume (Sieb.) Sieb.et Zucc., Smoked plum, 乌梅
Morus alba L., fruit, 桑椹子
Enhydris chinensia (Gray), Chinese Water snake skin, 玳瑁
Copper, Pyrite, 自然铜
Allium fistulosum L., Fistular Onion bulb, 葱白
Pharbitis purpurea (L.) Voight, Pharbitis seed, 牵牛子
Eriobotrya japonica (Thunb.) Lindl., Loquat leaf, 枇杷叶
Passiflora cochinchinensis Spreng., all-grass of Cochinchina Passionfower, 蛇王藤
Ulva lactuca L., algae, 石莼
Silybum marianum (L.) Gaertn., all-grass of St. Marys, 水飞蓟
Pleurotus ostreatus (Jacg:Fr.)Kummer, mushroom, 侧耳
Panax ginseng C.A.Mey., Ginseng root, 人参
Zea mays L., Corn Stigma, 玉米须
Pueraria lobata (Willd.) Ohwi, root of Lobed kudzuvine, 葛根
Brassica oleracea L.Var.Capitata L., stem an leaf of Cabbage, 甘蓝
Saccharum sinensis Roxb., Sugarcane, 甘蔗
Glycyrrhiza uralensis Fisch., tip of Licorice root, 甘草梢
Haliplanella luciae(Verrill), whole plant, 纵条肌海葵
Gentianopsis paludosa (Mum.) Ma, all grass, 湿生扁蕾

Solanum melongena L., fruit of Garden Eggplant, 茄子

Mytilus crassitesta Lischke, Mussel, 淡菜

Viscum coloratum (Komar.) Nakai, wig, 槲寄生

Aloe vera L., Aloe, 芦荟

Trichosanthes kirilowii Maxim, Snakegourd peel, 栝楼皮

Begonia semperflorens-hybr., all grass, 四季海棠

Rehmannia glutinosa Libosch., rhizome of Adhesive Rehmannia, 干地黄

Oryza Sativa L., Rice, 粳米

Ginkgo biloba L., Ginkgo leaf, 白果叶

Xanthium sibircium Patr. ex Widd., fruit of Siberian Cocklebur, 苍耳

Astragalus complanatus R. Brown, seed, 沙苑蒺藜

Rhodiola henryi (Diels) S.H.Fu, root, 豌豆七根

Melilotus suaveolens Ledeb., all grass, 白花辟汗草

Arachis hypogaea L., seed skin, 花生衣

Rauvolfia serpentina (L.) Benth. et Hook. f., root and bark and leaf, 蛇根木

Citrus aurantium L., immature fruit of Trifoliate-orange, 枳实

Cordyceps sinensis (Bark.). Sacc., Chinese Caterpillar fungus, 冬虫夏草

Quercus acutissima Carr., fruit of Sawtooth Oak, 橡实

Peganum harmala L., all-grass of Common peganum, 骆驼蓬

Lucuma nervosa A.DC., fruit of Eggfruit, 鸡蛋果

Cordyceps hawkesii Gray, fungus, 亚香棒虫草

Amaranthus lividus L., seed, 野苋子

Viscum multinerve (Hayata), wig, 柄果槲寄生

panax pseudoginseng Wall. Var. Japonicus (C.A.Mey) Hoo & Tseng, rhiome of Largelaef Japanese Ginseng, 珠儿参

Piper longum L., Long peppeer, 荜茇

Rhododoendron mucronulatum Turcz., leaf of Korean Rhododoendron, 迎山红

Psidium guajava L., leaf of Guava, 番石榴叶

Skimmia reevesiana Fortune, stem and leaf of Reeves skimmia, 茵芋

Amentotaxus argotaenia (Hance) Pilger, seed, 穗花杉种子

Hemerocallis plicata Stapf., root of Foldleaf Daylily, 黄花菜

Helianthus annuus L., Sunflower leaf, 向日葵叶

Thespesia populnea (L.) Soland.ex Corr, whole plant, 伞杨

Hedysarum polybotrys Hand.-Mazz., root of Manyinflorescenced Sweetvetch., 红芪

Vinegar, 醋

Trifolium pratente L., all-grass of Red clover, 红车轴草

Rubus adenophorus Rolfe, root, 红牛毛刺根
Tulipa gesneriana L., flower of Common Tulip, 郁金香
Camellia sinensis Kuntze, Tea leaf, 茶叶
Misgurnus anguillicaudatus (Cantor), Oriental weatherfish, 泥鳅
Arachis hypogaea L., seed oil, 花生油
Prunus armenica L.var.ansu Maxim., fruit of Apricot, 杏子
Mimosa pudica L., all-grass of Sensitiveplant, 含羞草
Juglans regia L., Walnut meat, 胡桃仁
Paederia scandens (Lour.) Merr., fruit of Chinese Fevervine, 鸡屎藤果
Allium sativum L., Crown's Treacle, 大蒜
Rosa rugosa Thunb., Rose, 玫瑰花
Rabdosia eriocalys (Dunn) Hara, leaf or root, 荷麻根
Imperata Cylindrica Beauv. var. major (Nees) C.E.Hubb., Leaf of LaLang Grass, 茅草叶
Litchi chinensis Sonn., Litchi seed, 荔枝核
Eriobotrya japonica (Thunb.) Lindl., Loquat fruit, 枇杷
Euphorbia antiquorum L., whole plant, 霸王鞭
Levisticum officinale Koch, root of Garden Lovage, 欧当归
Dryopteris filix-mas (L.) Schott, rhizome, 欧绵马
Polygonum cuspidatum Sieb.et Zucc., leaf of Giant Knotweed, 虎杖叶
Citrus junos Tanaka, fruit of Fragrant citrus, 橙子
Cytisus scoparius Link, all-grass of Scotch Broom, 金雀儿
Zanthoxylum podocarpum Hemsl., root-bark of Stalkedfruit Pricklyash., 麻口皮子药
Foeniculum vulgare Mill., stem and leaf of Fennel, 茴香茎叶
Gossypium herbaceum L., root of Levant Cotton, 棉花根
Citrus medica L., Citron fruit, 香橼
Poncirus trifoliata (L.) Raf., seed, 枸橘该
Diospyros kaki L.f., calyx and receptacle of a persimmon, 柿蒂
Diospyros kaki L.f., leaf of Persimmon, 柿叶
Allium tuberosum Rottl.ex Spreng, root, 韭根
Scepteridium ternatum, all grass, 独脚金鸡
Cinnamomum Camphora (L.) Presl., bark of Camphortree, 樟树皮
Morus alba L., Mulberry leaf, 桑叶
Prunus persica (L.) Batsch, Peach., 桃子
Corbicula fluminea (Muller), freshwater small clam, 蚬肉
Clerodendron bungei Steud., stem and leaf of Rose Glorybower, 臭牡丹

Tamarindus indica L., fruit of Tamarind, 酸角
Citrus tangerina Hort et Tanaka, Tangerine, 橘
Melilotus officinalis (L.) Desr., all grass, 黄零陵香
Stevia rebaudiana (Bertoni) Hemsl, leaf, 甜叶菊
Aioe arborescens Mill.var.natalensis Berger, juicy leaf, 鹿角芦荟
Macaca mulatta Zimmmermann, rhesus monkey, 猕猴肉
Actinidia arguta (Sieb.et Zucc.) Flarich.ex Miq., root, 猕猴梨根
Ranarugosa (schlegel)., toad, 粗皮蛙
Maytenusconfertflora J.Y. Luo et XX Chen., leaf, 密花美登木
Vitis vinifera L., Stem and leaf of European Grape, 葡萄藤叶
Cocos nucifera L., juice and shell, 椰子
Albizzia julibrissin Durazz., bark, 大叶合欢皮
Apis cerana Fabr, Bee Propolis, 蜂胶
Rehmannia glutinosa (Gaertn.) Libosch., prepared rhizome of Adhesive Rehmannia, 熟地黄
Fermented milk, 醍醐
Extract of beef, 霞天膏
Panax ginseng C.A.Mey., Ginseng fruit, 人参子
Murraya exotica L., root of Common Jasminorange, 九里香根

Table S5 HAT-interacting TCM medicinals

HAT
Harengula zunasi (Bleeker), Pilchard, 青鳞鱼
Eupatorium cannabinum L., all grass, 大麻叶佩兰
Securinega suffruticosa (Pall.) Rehd., twig and leaf of suffrutesent Securinega, 一叶萩
Artemisia capillaris Thunb., seedling of Capillary Wormwood, 茵陈蒿
Carica papaya L., Papaya, 番木瓜
Imperata cylindrica Beauv.var.major (Nees) C.E.Hubb., rhizome of Lalang Grass, 白茅根
Agrimonia pilosa Ledeb., all-grass of Hairyvein Agrimonia, 仙鹤草
Equisetum arvense Linn., all grass, 问荆
Polygonum multiflorum Thunb., root of Tuber Fleeceflower, 何首乌
Camellia japonica L., flower of Japanese camellia, 山茶花
Polygonum bistorta L., rhizome of Bistort, 拳参
Bupleurum komarovianum Lincz., root of Siberian Thorowax, 柴胡
Rhus succedanea L., root and leaf and bark and fruit, 野漆树
Panax notoginseng (Burk.) F.H.Chen, Sanchi, 三七
Angelica sinensis (Oliv) Diels., root of Chinese Angelica, 当归
Paeonia lactiflora Pall.; Paeonia veitchii Lynch, Radix Paeoniae Rubra, 赤芍
Phyllanthus urinaria L., whole plant, 叶下珠
Moschus sifanicus Przewalski, Musk, 麝香
Arisaema elephas S.Buchet, tuber of korean Jackinthepulpit, 天南星
Potentilla discolor Bunge, all-grass of Discolor Cinquefoil, 翻白草
Platycarya strobilacea Sieb.et Zucc., Leaf of Roundfruit Dyetree, 化香树叶
Paris polyphylla var.chinensis, petiolata Parus Rhizome, 蚤休
Cocculus laurifolius DC., root of Laurelleaf snailseed, 衡州乌药
Ampelopsis japonica (Thunb.) Makino, root of Japanese Ampelopsis, 白蔹
Potentilla chinensis Ser., all-grass of Chinese Cinquefoil, 委陵菜
Bauhinia championii (Benth.) Benth., stem of Champion Bauhinia, 九龙藤
Ligusticum chuanxiong Hort., rizome of Chuanxiong Ligusticum, 川芎
Polygonum chinense L., all-grass of Chinese Knotweed, 火炭母草
Ampelopsis brevipedunculata (Maxim.) Trautv., root of Amur Ampelopsis, 蛇葡萄根
Scolopendra subspinipes mutilans L.Koch., Centipede, 蜈蚣
Coriolus versicolor (Lr.) Quel, Polystictus versicolor (L.) Fr., 云芝
Euphorbia humifusa Willd., all-grass of Humifuse Euphorbia, 地锦草

Aconitum carmichaeli Pcbx, rhizome, 川乌头
Rheum palmatum L., root and rhizome of Sorrel Rhubarb, 大黄
Coix lacryma-jobi L.var.monilifer Watt, root, 川谷根
Polygonum hydropiper L., all-grass of Red-knees, 水蓼
Acanthopanax giraldii Harms, bark of Girald Acanthopanax, 红毛五加皮
Anthriscus sylvestris (L.) Hoffm., root of Woodland Beakchervil, 峨参
Ligustrum lucidum Ait., fruit of Glossy privet, 女贞子
Paederia scandens (Lour.) Merr., all-grass of Chinese Fevervine, 鸡屎藤
Acer ginnala Maxim., bud, 桑芽
Homo, Boys'urine (Urine of Boys under 10), 人尿
Abrus precatorius L., seed of Coralhead Plant, 相思子
Trogopterus xanthipes Milne-Edwards, Trogopterus dung, 五灵脂
Vallisneria spiralis L., all-grass of Spiral Wildcelery, 苦草
Polygonum capitatum Ham.ex D.Don, all-grass of Pinkhead knotweed, 石莽草
Phyllanthus amarus Schum. & Thonn., leaf, 小返魂
Duchesnea indica (Andr.) Focke., all-grass of Indian Mockstrawberry, 蛇莓
Typha angustifolia L., pollen of Longbract Cattail., 蒲黄
Cetacea, whale, 鲸肉
Liticauda semifasciata (Reinhardt), Erabu-unagi, 蛇婆
Acalypha australis L., all-grass of Copperleaf, 铁苋
Gnetum parvifolium (Warb.) C.Y.Cheng, Stem of Smallleaf Jointfir, 买麻藤
Euphorbia hirta L., all-grass of Garden Euphorbia, 大飞扬草
Pegasus laternarius (cuvier), whole fish, 海蛾
Acer davidii Franch., leaf of David Maple, 青榨槭
Mugil cephalus L., Striped mullet, 鲻鱼
Passiflora caerulea L., all-grass of Passionflower, 西番莲
Cervus nippon Temminck, Pilose Anter, 鹿茸
Casuarina equisetifolia .Forst., fresh leaf or bark, 木麻黄
Euphorbia helioscopia L., all-grass of Sun Euphorbia, 泽漆
Euonymus bungeanus, root and bark and leaf, 丝棉木
Astragalus membranaceus (Fisch.) Bge. Var. Monghol, Radix Astragali, 黄芪
Hemidactylus, Gommon House Gecko, 蜥虎
Saxifraga stolonifera (L.) Meerb., all-grass of Creeping Rockfoil, 虎耳草
Erodium stephanianum Willd., all-grass of Common Heron's bill, 老鹳草
Malus pumila Mill., Apple, 苹果

Vespertilio superans Thomas, Bat dung, 夜明砂
Senecio chrysanthemoides DC., all-grass of Chrysanthemum-like Groundsel, 土三七
Rubus innominatus S. Moors, root, 白叶莓
Equisetum hiemale L., horsetail, 木贼
Insect gall, Nutgalls, 没食子
Eriocheir sinensis H.Milne-Edwards, Carb, 蟹
Mamta birostris (walbaum), gill, 蝠鲼鳃
Acer truncatum Bunge, root-bark of Purpleblow Maple, 元宝槭
Paeonia suffruticosa Andr., Tree Peony bark, 牡丹皮
Ganoderma lucidum, Lingzhi mushroom, 灵芝
Gossampinus malabarica (DC.) Merr., flower of Common Bombax, 木棉花
Abutilon indicum (L.) Sweet, all-grass of Indian Abutilon, 磨盘草
Acanthus ilicifolius L, whole plant, 老鼠簕
Catharanthus roseus (L.) G.Don, all-grass of Madagascar Periwinkle, 长春花
Rhus chinensis Mill, root, 盐肤木根
Tremella fuciformis Berk, fruiting body, 银耳
Euphorbia nematocypha Hand.Mazz., root of Yunnan Euphorbia, 大狼毒
Tetraplodon mnioides (Hedw.) B.S.G., moss, 并齿藓
Penaeus orientalis Kish., lobster, 对虾
Cabomba caroliniana, Axle Algae, 鱼草
Urtica macrorrhiza Hand.-Mazz., whole plant, 青活麻
Coprinus sterqulinus Fr., fruiting body, 鬼盖
Mustelus manazo Bleeder, shark, 鲨鱼肉
Lycium dasystemum pojark., fruit of Hairystamen Wolfberry, 枸杞子
Anguilla japonica Temminck et Schlegel, Japanese eel, 鳗鲡鱼
Bubalus bubalis L., Ox blood, 牛血
Bubalus bubalis L., milk, 牛乳
Bubalus bubalis L., Ox gallbladder, 牛胆
Phyllanthus emblica L., fruit of Emblic leafflower, 余甘子
Microsorium fortune (Moore) Ching, all grass and rhizome, 大叶骨牌草
Sarcodon fuligineo-albus (Fr.) Quel., fruiting body, 褐盖肉齿菌
Typhonium giganteum Engl., rhizome, 白附子
Antenoron neofiliforme (Nakai) Hara., root of Shorthairy Antenoron, 金线草根
Copyindscomatus (MUII.Fr) Gray, mushroom, 鸡腿蘑
Eucalyptus citriodora, leaf, 柠檬桉叶

Rana nigromaculata Hallowell, Pond green frog, 青蛙
Glyptostrobus pensilis, bark and leaf, 水松
Rose chinensis Jacq., flower of Chinese Rose, 月季花
Nicotiana tabacum L., leaf of Common Tobacco, 烟草
Ginkgo biloba L., seed of Maidenhairtree, 白果
Glycine max.(L.) Merr., black seed of Soybean., 黑大豆
Pharbitis purpurea (L.) Voight, Pharbitis seed, 牵牛子
Marchantia polymorpha L., all grass, 地钱
Agaricus campestris L.ex Fr., Meadow mushroom Mushroom, 蘑菇
Ulva lactuca L., algae, 石莼
Homonoia riparia Lour., root of Riparian Homonoia, 水杨柳
Phragmites communis Trin., Reed rhizome, 芦根
Polygonum hydropiper L., root of Red-Knees, 水蓼根
Glechoma Longituba (Nakai) Kupr., all-grass of Longtube Ground Ivy, 金钱草
Panax ginseng C.A.Mey., Ginseng root, 人参
Bixa orellana, bark and leaf and fruit and seed, 胭脂木
Colysis hemionitidea (Wall.) Presl., leaf, 断线蕨
Asplenium trichomanes L., all grass, 铁角风尾草
Rose multiflora Thunb., fruit of Japanese Rose, 营实
Artemisia sphaerocephala Krasch., all-grass of Roundhead Wormwood., 白沙蒿
Aconitum carmichaeli Debx., Prepared lateral root of Common Monkshood., 附子
Ginkgo biloba L., Ginkgo leaf, 白果叶
Homo, urine, 人中白
Rhodiola henryi (Diels) S.H.Fu, root, 豌豆七根
Cordyceps sinensis (Bark.). Sacc., Chinese Caterpillar fungus, 冬虫夏草
Rhodiola sacra (Hamet) S.H.Fu., all-grass of Integripetal Rhodiola, 红景天
Lucuma nervosa A.DC., fruit of Eggfruit, 鸡蛋果
Quercus dentata Thunb., bark of Daimyo Oak., 槲皮
Rhododoendron mucronulatum Turcz., leaf of Korean Rhododoendron, 迎山红
Mentha rotundifolia (L.) Huds., all-grass of Apple Mint, 鱼香草
Trifolium pratente L., all-grass of Red clover, 红车轴草
Camellia sinensis Kuntze, Tea leaf, 茶叶
Misgurnus anguillicaudatus (Cantor), Oriental weatherfish, 泥鳅
Prunus armenica L.var.ansu Maxim., fruit of Apricot, 杏子
Cantharellus cibarius Fr., fruiting body, 鸡油菌

Myrica rubra (Lour.) Sieb.et Zucc., bark of Chinese Waxmyrtle, 杨梅树皮
Juglans regia L., Walnut meat, 胡桃仁
Phaseolus radiatus L., Mung Bean, 绿豆
Eriobotrya japonica (Thunb.) Lindl., Loquat fruit, 枇杷
Hoplobatrachus tigerinus, tiger frog, 虎纹蛙
Chelomia mydas (Linnaeus)., sea turtle, 海龟
Juglans regia L., leaf of English Walnut, 胡桃叶
Cinnamomum Camphora (L.) Presl., bark of Camphortree, 樟树皮
Morus alba L., Mulberry leaf, 桑叶
Castanea mollissima Bl., leaf of Hairy Chestnut, 栗叶
Castanea mollissima Bl., bark of Hairy chesstnut, 栗树皮
Castanea mollissima Bl., root of Hairy chestnut, 栗树根
Corbicula fluminea (Muller), freshwater small clam, 蚬肉
Cotinus coggygria Scop., leaf, 黄栌枝叶
Agaricus campestris, wild mushroom, 野蘑菇
Cervus elaphus L., Refuse of Deerhorn glue, 鹿角霜
Ranarugosa (schlegel)., toad, 粗皮蛙
Ricinus communis L., Castor leaf, 蓖麻叶
Polygonum divaricatum L., root of Divaricate Knotweed, 酸不溜根
Potentilla anserina L., all grass, 蕨麻草
Rehmannia glutinosa (Gaertn.) Libosch., prepared rhizome of Adhesive Rehmannia, 熟地黄
Fermented milk, 醍醐
Extract of beef, 霞天膏
Scoparia dulcis L., whole plant, 野甘草
Aconitum carmichaeli Debx., Prepared lateral root of Common Monkshood., 附子

Table S6 HMT-interacting TCM medicinals

HMT
Inula japonica Thunb., flower, 旋覆花
Tupistra ensifolia Wang et Tang, rhizome, 岩七
Passiflora foetida L., all-grass or fruit of Tagua Passionflower, 龙珠果
Cynanchum bungei Decne., root of Bunge Swallowwort, 白首乌
Scleroderma bovista Fr., fruiting body, 硬皮马勃
Phoenix dactylifera L., Date, 无漏子
Artemisia capillaris Thunb., seedling of Capillary Wormwood, 茵陈蒿
Carica papaya L., Papaya, 番木瓜
Capsella brusa-pastoris (L.) Medic., Herba Capsellae, 荠菜
Smilax lanceifolia var. opace A.DC., rhizome of Bluebead Greenbrier, 土茯苓
Lilium cernuum Kom., bulb of Low Lily, 百合
Polygonum multiflorum Thunb., root of Tuber Fleeceflower, 何首乌
Ipomoea batatas (L.) Lam., Sweet otato, 番薯
Armeniaca vulgaris Lam., Apricot seed, 杏仁
Gardenia jasminoides Ellis, fruit of Cape Jasmine, 栀子
Prunus persica (L.) Batsch, Peach kernel., 桃仁
Bupleurum komarovianum Lincz., root of Siberian Thorowax, 柴胡
Averrhoa carambola L., fruit of Common Averrhoa, 阳桃
Panax notoginseng (Burk.) F.H.Chen, Sanchi, 三七
Angelica sinensis (Oliv) Diels., root of Chinese Angelica, 当归
Ilex rotunda Thunb., bark of Ovateleaf Holly, 救必应
Veronicastrum sibiricum (L.) Pen-nell., root and all grass, 草本威灵仙
Myrica rubra (Lour.) Sieb.et Zucc., fruit of Chinese Waxmyrtle, 杨梅
Gleditsia sinensis Lam., spine of Chinese Honeylocust, 皂角刺
Sanguisorba officinalis var.longifila (Kitag) Yu.et Li, root of Garden Burnet, 地榆
Vaccinium vitis-idaea L., Cowberry leaf, 越橘叶
Cornus officinalis Sieb. et Zucc., fruit of Asiatic Cornelian cherry, 山茱萸
Ampelopsis brevipedunculata (Maxim.) Trautv., root and bark, 蛇白蔹
Selaginella moellendorfii Hieron., all-grass of Moellendorf's Spikemoss, 地柏枝
Sloanum nigrum L., all-grass of Black Nightshade, 龙葵
Phytolacca acinosa Roxb., root of Indian Pokeweed, 商陆
Dipsacus asper Wall., root of Himalayan Teasel, 续断

Daphne odora Thunb., flower of Winter Daphne, 瑞香花
Polygonum chinense L., all-grass of Chinese Knotweed, 火炭母草
Swertia punicea Hemsl., all-grass of Scarlet swerita, 山飘儿草
Camptotheca acuminata Decne., fruit, 喜树
Epimedium acuminatum Franch., all-grass of Longspur Epimedium, 淫羊藿
Coriolus versicolor (Lr.) Quel, Polystictus versicolor (L.) Fr., 云芝
Rheum palmatum L., root and rhizome of Sorrel Rhubarb, 大黄
Agastache rugosus (Fisch. et Mey) O Ktze., all-grass of Wrinkled Gianthyssop, 藿香
Lysimachia foenum-graecum Hance, whole plant, 灵香草
Conyza bonariensis (L.) Cronq., whole plant, 野塘蒿
Trogopterus xanthipes Milne-Edwards, Trogopterus dung, 五灵脂
Salvia trijuga Diels, root, 三对叶丹参
Oenanthe javanica (Bl.) DC., all-grass of Javan Waterdropwort, 水芹
Cassia tora L., seed of Sickle Senna, 决明子
Populus pseudosimonii Kitag., bark of False simon poplar, 小青杨
Duchesnea indica (Andr.) Focke., all-grass of Indian Mockstrawberry, 蛇莓
Endarachne binghamiae J.Ag., algae, 鹅肠菜
Arenaria serpyllifolia L., all-grass of Creepingthymeleaf sandwort, 小无心菜
Asterina pectinifera Miller et Troschel, Petrel, 海燕
Polypodium vulgare L., Polypodium virginianum L., rhizome, 多足蕨
Morus australis Poir., root, 鸡桑根
Sparganium stoloniferum Buch.-Ham., rhizome of Common Burreed, 三棱
Blatta orientalis L., Oriental Cockroach, 蟑螂
Spinacia oleracea L., all-grass of Spinach, 菠菜
Euphorbia helioscopia L., all-grass of Sun Euphorbia, 泽漆
Aesculus chinensis Bunge var.chekiangensis (Hu et Fran) Fang, seed of Chinese Buckeye, 娑罗子
Elephantopus scaber L., all-grass of Scabrous Elephantfoot, 苦地胆
Astragalus membranaceus (Fisch.) Bge. Var. Monghol, Radix Astragali, 黄芪
Apis cerana Fabricius, Royal jelly, 蜂乳
Malus pumila Mill., Apple, 苹果
Ziziphus jujuba Mill, Chinese date, 大枣
Eriocheir sinensis H.Milne-Edwards, Carb, 蟹
Uncaria hirsuta Havil, Gambirplant stem with hooks., 钩藤
Glycyrrhiza uralensis Fisch., root of Glabrousfruit Licorice, 甘草
Ganoderma lucidum, Lingzhi mushroom, 灵芝

Medicago sativa L., all grass, 苜蓿
Alnus japonica (Thunb) Steud., twig and leaf of Japanese Alder, 赤杨
Ganoderma applanatum (Pers.ex Wallr.) Pat., fruiting body, 树舌
Lanminaria japonica Aresch., Kelp, 昆布
Philosamia cynthia ricini (Donovan), larvae, 蓖麻蚕
Polygonum perfoliatum L, whole plant, 扛板归
Tremella fuciformis Berk, fruiting body, 银耳
Euphorbia nematocypha Hand.Mazz., root of Yunnan Euphorbia, 大狼毒
Poria cocos (Schw.) Wolf., Indian Bread, 茯苓
Pseudostellaria heterophylla (Miq.) Pax ex Pax et , Radix Pseudostellariae, 太子参
Lycium dasystemum pojark., fruit of Hairystamen Wolfberry, 枸杞子
Apis mellifera, Honey, 蜂蜜
Anguilla japonica Temminck et Schlegel, Japanese eel, 鳗鲡鱼
Bubalus bubalis L., Ox liver, 牛肝
Stauntonia hexaphylla (Thunb.) Decne., stem and root, 牛藤
Xanthium sibircium Patr. ex Widd., fruit of Siberian Cocklebur, 苍耳子
Rhaponticum uniflorum (L.) DC., root, 漏芦
Rana nigromaculata Hallowell, Pond green frog, 青蛙
Glyptostrobus pensilis, bark and leaf, 水松
Amaranthus ascendens Loisel., all grass and root, 野苋菜
Drymaria cordata (L.) Willd., all-grass of West Indian chickweed, 荷莲豆菜
Prunus mume (Sieb.) Sieb.et Zucc., Smoked plum, 乌梅
Morus alba L., fruit, 桑椹子
Delongix regia (Boj.) Raf., twig and leaf of Flamboyantree, 凤凰木
Tripterygium hypoglaucum (Devl.) Hutch., root, 昆明山海棠
Cannabis sativa L., Hemp leaf, 麻叶
Polygonum chinense L., root of Chinese Knotweed, 火炭母草根
Zacco platypus (Temminck et Schlegel), pale chub, 石鲋鱼
Ulva lactuca L., algae, 石莼
Pleurotus ostreatus (Jacg:Fr.)Kummer, mushroom, 侧耳
Juncus effusus L., Rush , 灯心草
Panax ginseng C.A.Mey., Ginseng root, 人参
Commiphora myrrha Engl., Myrrh, 没药
Ilex cornuta Lindl.ex Paxt., leaf, 功劳叶
Enteromorpha Linza (L.) Ag., algae, 干苔

Balanophora japonica Makino, all-grass of Japanese Balanophora, 葛蕈
Tripterygium regeli Sprague et Tak., root of Regel Threewingnut, 东北雷公藤
Pteris fauriei Heiro., leaf, 金钗凤尾蕨
Viscum coloratum (Komar.) Nakai, wig, 槲寄生
Platycodon grandiflorum (Jacq.) A.DC., Balloonflower root, 桔梗
Homo, Dried Human placenta, 紫河车
Euphorbia tirucalli, all grass, 绿玉树
Melilotus suaveolens Ledeb., all grass, 白花辟汗草
Biota orientalis (L.) Endl., leafy twigs of Chinese Arborvitae, 侧柏叶
Quercus acutissima Carr., fruit of Sawtooth Oak, 橡实
Amaranthus tricolor L., all-grass of Threecoloured Amaranth, 苋
Peganum harmala L., all-grass of Common peganum, 骆驼蓬
Amaranthus lividus L., seed, 野苋子
Crocus sativus L., stigma, 番红花
Halenia corniculata (L.) Cornaz., all-grass of Corniculate spurgentian, 花锚
Viscum multinerve (Hayata), wig, 柄果槲寄生
Hypocrella bambusae (Berk.et Br.) Sacc., mushroom, 竹砂仁
Eupatorium chinense L., root, 华泽兰
Lycopodium annotinum L., all grass, 杉蔓石松
Marasmius androsaceus (L .ex Fr.) Fr., fungus, 鬼毛针
Trifolium pratente L., all-grass of Red clover, 红车轴草
Fomitopsis pinicola (Sow.ex Fr.) Karst., fruiting body, 红缘层孔菌
Boehmeria tricuspis (Hance) Makino, leaf or root of Tricuspidate Falsenettle, 赤麻
Misgurnus anguillicaudatus (Cantor), Oriental weatherfish, 泥鳅
Cantharellus cibarius Fr., fruiting body, 鸡油菌
Myrica rubra (Lour.) Sieb.et Zucc., bark of Chinese Waxmyrtle, 杨梅树皮
Mimosa pudica L., all-grass of Sensitiveplant, 含羞草
Picrasmaquassioides (D.Don) Benn., root, 苦木根
Physalis pubescens L., fruit, 苦蘵果实
Rabdosia eriocalys (Dunn) Hara, leaf or root, 荷麻根
Viburnum dilatatum Thunb., stem and leaf of Linden Viburnum, 荚蒾
Dryopteris filix-mas (L.) Schott, rhizome, 欧绵马
Achillea alpina L., all-grass of Alpine Yarrow, 一枝蒿
Codiaeum variegatum (Linn), leaf, 洒金榕
Peganum nigellastrum Bunge, all grass, 骆驼蒿

Abrus precatorius, root, 相思子根
Diospyros kaki L.f., Colloidal liquid produced by immature Persimmon, 柿漆
Thuja occidentalis L., leaf and wood, 香柏
Phytolacca acinosa Roxb., seed, 美商陆子
Morus alba L., Mulberry leaf, 桑叶
Anthriscus syluestris (L.) Hoffm., leaf, 峨参叶
Phoca vitulina, oil, 海豹油
Melilotus officinalis (L.) Desr., all grass, 黄零陵香
Stevia rebaudiana (Bertoni) Hemsl, leaf, 甜叶菊
Ranarugosa (schlegel)., toad, 粗皮蛙
Amorpha fruticosa L., root, 紫穗槐
Panax ginseng C.A.Mey., Ginseng fruit, 人参子

Table S7 MBD-interacting TCM medicinals

MBD
Potentilla freyniana Bornm, root or all grass, 地蜂子
Clematis armandii Franch., stem of Armand clematis, 川木通
Tupistra ensifolia Wang et Tang, rhizome, 岩七
Alstonia scholaris (L.) R.Br., bark of Common Alstonia, 象皮木
Ajuga macrosperma Wall. Ex Benth., all grass, 拔毒草
Epilobium hirsutum L., flower and root, 柳叶菜
Cynanchum bungei Decne., root of Bunge Swallowwort, 白首乌
Harengula zunasi (Bleeker), Pilchard, 青鳞鱼
Eupatorium cannabinum L., all grass, 大麻叶佩兰
Securinega suffruticosa (Pall.) Rehd., twig and leaf of suffrutesent Securinega, 一叶萩
Helianthus annuus L., seed, 向日葵子
Akebia trifoliata (Thunb.) Koidz.var.australis (Diels) Rehd., Stem of Threeleaf Akebia, 木通
Phoenix dactylifera L., Date, 无漏子
Lycopodium japonicum Thunb., all grass, 伸筋草
Asparagus cochinchinensis (Lour.)Merr., root of Cochinchinese Asparagus, 天门冬
Polygala wattersii Hance, root of Watter Millkwort, 山桂花
Ilex chinensis Sims, leaf, 四季青
Carica papaya L., Papaya, 番木瓜
Rhododendron micranthum Turcz., leaf or flower of Manchurian Rhododendron, 照山白
Rauvolfia verticillata (Lour) Baill., root of Common Devilpepper, 萝芙木
Sphenomeris chinensis (L.) Maxon, all grass, 大叶金花草
Equisetum arvense Linn., all grass, 问荆
Trionyx sinensis Wiegmann, turtle shell, 鳖甲
Zanthoxylum nitidum (Roxb.) DC., root of Shinyleaf Pricklyash, 入地金牛
Capsella brusa-pastoris (L.) Medic., Herba Capsellae, 荠菜
ParisverticillataM.Bieb., rhizome, 上天梯
Codonopsis tubulosa Kom., root of Pilose Asiabell, 党参
Manis pentadactyla Linnaeus, Pangolin scales, 穿山甲
Dioscorea opposita Thunb., rhizome of Common Yam, 山药
Dicranopteris pedata (Houtt.) Nakai, leaf, 芒萁骨
Prunus japonica Var. nakai (Levl.) Rehd., seed of Downy cherry, 郁李仁
Tephrosia purpurea (L.) Pers., root and stem and leaf, 灰叶

Ziziphus jujuba var spinosa (Bunge) Hu, seed of Spine Date, 酸枣仁
Camellia japonica L., flower of Japanese camellia, 山茶花
Gardenia jasminoides Ellis, fruit of Cape Jasmine, 栀子
Polygonum bistorta L., rhizome of Bistort, 拳参
Rubus corchorifolius L. F., root and leaf, 山莓
Bupleurum komarovianum Lincz., root of Siberian Thorowax, 柴胡
Allium victorialis, wild onion, 茖葱
Cryptotympana pustulata Fabricius, Cicada slough, 蝉蜕
Panax notoginseng (Burk.) F.H.Chen, Sanchi, 三七
Angelica sinensis (Oliv) Diels., root of Chinese Angelica, 当归
Buthus martensii Karsch., Scorpion, 全蝎
Psychotria rubra (Lour.) Poir., tuig and leaf of Red psychotria, 山大刀
Asparagus filicinus, root, 羊齿天冬
Clematis apiifolia DC., stem of October clematis, 女萎
Clematis armandii Franch., root of Chinese clematis, 威灵仙
Pteridium aquilinum (L.) Kuhn var.latiusculum (Desv.) Underw, all-grass of Eastern Bracken Fern, 蕨
Rosa laevigata Michx., Cherokee Rose Fruit, 金樱子
Sargentodoxa Cuneata (oliv.) Rehd. et wils., Stem of Sargentgloryvine, 大血藤
Arisaema elephas S.Buchet, tuber of korean Jackinthepulpit, 天南星
Polygonum cuspidatum Sieb.et Zucc., rhizome of Giant Knotweed, 虎杖
Vaccinium vitis-idaea L., Cowberry leaf, 越橘叶
Potentilla discolor Bunge, all-grass of Discolor Cinquefoil, 翻白草
Trichosanthes kirilowii Maxim, fruit of Mongolian Snakegourd, 栝楼
Crotalaria sessiliflora Linn., all grass, 农吉利
Photinia serrulata Lindl., leaf, 石南
Bidens bipinnata L., all-grass of Spanishneedles, 鬼针草
Cornus officinalis Sieb. et Zucc., fruit of Asiatic Cornelian cherry, 山茱萸
Scutellaria rehderiana Diels, root of Baikal skullcap, 黄芩
Ampelopsis brevipedunculata (Maxim.) Trautv., root and bark, 蛇白蔹
Centipeda minima (L.) A.Br. et Aschers., all-grass of Small Centipeda, 鹅不食草
Solanum tuberosum L., potato, 马铃薯
Anemarrhena asphodeloides Bunge, rhizome of Common Anemarrhena, 知母
Spilanthes acmella (L.) Dalz.et Gibs., all-grass of Paniculate spotflower, 天文草
Stellera chamaejasme L., root, 狼毒
Changium smyrnioides Wolff, root of Medicinal Changium, 明党参

Rhododendron dauricum L., leaf of Dahurian Rhododendron, 满山红
Scutellaria scordifolia Fisch, all grass, 头巾草
Ephedra aquisetina Bunge, stem of Chinese Ephedra, 麻黄
Ligusticum chuanxiong Hort., rizome of Chuanxiong Ligusticum, 川芎
Datura inoxia Mill., seed of Hindu Datura, 曼陀罗子
Aconitum coreanum (Levl) Raipaics, root of Korean Monkshood, 关白附
Trollius macropetalus Fr.Schmidt, flower, 长瓣金莲花
Pyrola rotundifolia L. subsp. chinensis H.Andres, , 鹿衔草
Photinia glabra (Thunb.) Maxim., leaf, 光叶石楠
Bletilla striata (Thunb.) Reichb.f., tuber of Common Bletilla, 白及
Thalictrum finetii Boivin, root and rhizome, 千里马
Scolopendra subspinipes mutilans L.Koch., Centipede, 蜈蚣
Cephalanoplos segetum (Bunge) Kitam., all-grass of Common cephalanoplos, 小蓟
Coriolus versicolor (Lr.) Quel, Polystictus versicolor (L.) Fr., 云芝
Oroxylum indicum (L.) Vent., seed of Indian Trumpetflower, 木蝴蝶
Aralia chinensis L, root skin and stem skin, 楤木
Artemisia anomala S.Moore, all-grass of Seleng Wormwood., 刘寄奴
Aconitum carmichaeli Pcbx, rhizome, 川乌头
Rheum palmatum L., root and rhizome of Sorrel Rhubarb, 大黄
Coptis teeta Wall., rhizome of Chinese Goldthread, 黄连
Zanthoxylum schinifolium Sieb.et Zucc., pericarp of Bunge pricklyash., 花椒
Melia azedarach L., bark of Szechwan chinaberry, 苦楝皮
Polygonum hydropiper L., all-grass of Red-knees, 水蓼
Cynanchum inamoenum (Maxim) Loes., root and rhizome of Unpleasant Swallowwort, 老君须
Acanthopanax giraldii Harms, bark of Girald Acanthopanax, 红毛五加皮
Thalictrum baicalense Turcz., root and rhizome of Baikal Meadowrue, 马尾连
Bull feather, Oxhide gelatin, 黄明胶
Anthriscus sylvestris (L.) Hoffm., root of Woodland Beakchervil, 峨参
Vladimiria souliei (Franch.)Ling, root of Common Aucklandia, 木香
Pheretima aspergillum (E. Perrier), dried body, 地龙
Panax quinquefolium L., American Ginseng, 西洋参
Ligustrum lucidum Ait., fruit of Glossy privet, 女贞子
Patrinia villosa Juss., all-grass of Dahurian Patrinia, 败酱
Setaria italica (L.) Beauv., seed of Foxtail Millet, 粟米
Homo, Boys'urine (Urine of Boys under 10), 人尿

Semen Dolichoris Album, White Hyacinth Bean, 白扁豆
Trogopterus xanthipes Milne-Edwards, Trogopterus dung, 五灵脂
Pyrrosia gralla (Gies) Ching, leaf of Stilted pyrrosia, 石韦
Veronica peregrina L., all grass, 仙桃草
Asparagus setaceus (Kunth) Jessop, all-grass of Setose Asparagus, 文竹
Millettia dielsiana Harms ex Diels., Stem of Suberect spatholobus, 鸡血藤
Populus pseudosimonii Kitag., bark of False simon poplar, 小青杨
Gynostemma pentaphyllum (Thunb.) Mak., all grass, 绞股蓝
Lycopersicon esculentum Mill., Tomato, 番茄
Ruta graveolens L., all-grass of Common Rue, 臭草
Typha angustifolia L., pollen of Longbract Cattail., 蒲黄
Allium macrostemon Bunge, bulb of Longstamen Onion, 薤白
Chlorella pyrenoidosa Chick., algae, 小球藻
Wisteria sinensis Sweet, stem and leaf of Chinese Wisteria, 紫藤
Rubus lambertianus Ser. ex DC., root and leaf, 高梁泡
Ficus pumila L., root and stem and leaf and fruit, 薜荔
Canis familiaris L., testis and penis of a Dog, 狗鞭
Tagetes patula L., all-grass of French Marigold., 孔雀草
Polypodium vulgare L., Polypodium virginianum L., rhizome, 多足蕨
Clematis terniflora DC. [C.paniculata Thunb.], root, 铜脚威灵仙
Bulbophyllum odoratissimum Lindl., all-grass of Denseflower Bulbophyllum, 果上叶
Lycopodium selago L., all-grass of Fir clubmoss, 小接筋草
Medicago lupulina L., all-grass of Black Medic., 老蜗生
Armillariella mellea (Vahl.ex Fr.) Karst., fruiting body, 蜜环菌
Lindernia crustacea (L.) F.Muell., all-grass of Brittle Falsepimpernel, 母草
Scutellaria barbata D.Don., all-grass of Barbed Skullcap, 半枝莲
Thalictrum thunbergii DC., root, 烟窝草
Pegasus laternarius (cuvier), whole fish, 海蛾
Oxya chinensis Thunb., locust, 蚱蜢
Blatta orientalis L., Oriental Cockroach, 蟑螂
Spinacia oleracea L., all-grass of Spinach, 菠菜
Periploca calophylla (Wight) Falc., Stem of Prettyleaf Silkvine, 乌骚风
Osmunda japonice Thunb., Japanese Flowering Fern Rhizome, 紫萁贯众
Bubalus bubalis L., penis, 牛鞭
Passiflora caerulea L., all-grass of Passionflower, 西番莲

Spisula solida (Linnaeus, 1758), shell, 珂
Cervus nippon Temminck, Pilose Anter, 鹿茸
Vicia sativa L., all-grass of Common vetch, 大巢菜
Thalictrum delavayi Franch., root and rhizome, 偏翅唐松草
Thalictrum foetidum L., root and rhizome, 香唐松草
Thalictrum javanicum Bl, root and rhizome, 羊不食
Casuarina equisetifolia .Forst., fresh leaf or bark, 木麻黄
Artemisia japonica Thunb., all-grass of Japanese Wormwood., 牡蒿
Caltha palustris L., root and leaf, 驴蹄草
Thalictrum alpinum L., root and rhizome of Alpine Meadowrue, 高山唐松草
Thalictrum petaloideum L., root of Petalformed Meadowrue, 瓣蕊唐松草
Boswellia bhaw-dajiana Birdw., Frankincense, 乳香
Anodonta woodiana (Lea), Pearl, 珍珠
Astragalus membranaceus (Fisch.) Bge. Var. Monghol, Radix Astragali, 黄芪
Physalis alkekengi L.ver.franchetii (Mast.) Mak., Franchet Groundcherry, 挂金灯
Hemidactylus, Gommon House Gecko, 蜥虎
Bombyx mori L., Beauveria bassiana (Bals.) Vuill. infection, 白僵蚕
Bombyx mori L., Silk moth, 原蚕蛾
Bambusa textilis Mc-Clure, CONCRETIO SILICEA BAMBUSAE, 竹黄
Pelargonium hortorum Bail., flower, 石蜡红
Saxifraga stolonifera (L.) Meerb., all-grass of Creeping Rockfoil, 虎耳草
Erodium stephanianum Willd., all-grass of Common Heron's bill, 老鹳草
Malus pumila Mill., Apple, 苹果
Senecio chrysanthemoides DC., all-grass of Chrysanthemum-like Groundsel, 土三七
Citrullus lanatus (Thurb.) Mansfeld, Pulp of a Watermelon, 西瓜
Rubus innominatus S. Moors, root, 白叶莓
Equisetum hiemale L., horsetail, 木贼
Ficus carica L., Fig, 无花果
Sapindus mukorossi Gaertn., seed of Chinese soapberry, 无患子
Sepiella maindroni Rochebrune, inner shell, 海螵蛸
Eriocheir sinensis H.Milne-Edwards, Carb, 蟹
Mamta birostris (walbaum), gill, 蝠鲼鳃
Sapindus mukorossi Gaertn., skin, 无患子皮
Veronica anagallis-aquatca L, all grass, 水苦荬
Lepidium apetalum Willd., seed, 葶苈子

Chinemys reevesii (Gray), Carapax Et Plastrum Testudinis, 龟甲
Phaseolus vulgaris, bean, 菜豆
Kopsia officinalis Tsiang et P.T.Li, fruit or leaf of Medicinal Kopsia, 柯蒲木
Xanthoceras sorbifolia Bunge, wood or twig and leaf of Shinyleaf Yellowhorn., 文冠果
Ganoderma lucidum, Lingzhi mushroom, 灵芝
Cinnamomum casia Presl., Cassiabarktree bark , 肉桂
Eucommia ulmoides Oliv., Eucommia bark, 杜仲
Medicago sativa L., all grass, 苜蓿
Osmanthus fragrans Lour., flower of Sweet osmanthus, 桂花
Alnus japonica (Thunb) Steud., twig and leaf of Japanese Alder, 赤杨
Gossypium herbaceum L., seed of Levant Cotton, 棉花子
Olea europaea L., fruit oil, 齐墩果
Lanminaria japonica Aresch., Kelp, 昆布
Philosamia cynthia ricini (Donovan), larvae, 蓖麻蚕
Abutilon indicum (L.) Sweet, all-grass of Indian Abutilon, 磨盘草
Stellaria media (L.) Cyr., all-grass of Chickweed, 繁缕
Artemisia argyi Levl. et Vant., leaf of Argy Wormwood, 艾叶
Phaseolus lunatus L., Sieve Bean., 金甲豆
Agkistrodon acutus (Guenther), Agkistrodon, 蕲蛇
Asterias rollestoni, sea star, 海盘车
Trollius chinensis Bunge, flower of Chinese Globeflower, 金莲花
Polygonum perfoliatum L, whole plant, 扛板归
Brassica rapa L., root of Turnip, 芜菁
Tremella fuciformis Berk, fruiting body, 银耳
Poria cocos (Schw.) Wolf., Indian Bread, 茯苓
Penaeus orientalis Kish., lobster, 对虾
Spirulina, algae, 螺旋藻
Dichroa febrifuga Lour., root of Antifebrile Dichroa, 常山
Vicia japonica A.Gray, all-grass of Japanese Vetch, 东方野豌豆
Stromateoides argenteus (Euphrasen), Silvery pomfret, 鲳鱼
Lycium dasystemum pojark., fruit of Hairystamen Wolfberry, 枸杞子
Anguilla japonica Temminck et Schlegel, Japanese eel, 鳗鲡鱼
Mentha haplocalyx Briq., all-grass of Wild Mint, 薄荷
Bubalus bubalis L., cow-bezoar, 牛黄
Achyanthes aspera L., all grass, 倒扣草

Cynanchum paniculatum (Bge.) Kitag., rhizome, 牛心朴
Microsorium fortune (Moore) Ching, all grass and root, 大叶骨牌草
Cassia fistula L., fruit, 婆罗门皂荚
Rubus alceaefolius Poir., root or leaf of Roughleaf Raspberry, 粗叶悬钩子
Sarcodon fuligineo-albus (Fr.) Quel., fruiting body, 褐盖肉齿菌
Rubus multibracteatus Levl.et vant., all-grass of Multibract Raspberry, 大乌泡
Typhonium giganteum Engl., rhizome, 白附子
Equus asinus Linnaeus, E-gelatin, 阿胶
Aristolochia mollisima Hance, rhizome or all-grass of Wooly Dutchmanspipe, 寻骨风
Davallia mariesii Moore ex Bak., rhizome, 海洲骨碎补
Indigofera hirsuta L., root of Hirsute Indigo, 毛木蓝
Lepisma saccharina L., silverfish, 衣鱼
Inula japonica Thunb., all-grass of Japanese Inula, 金沸草
Wrightza tomentosa(Roxb.)Roem.et schult., root or stem, 胭木
Datura metet L., leaf of Hindu Datura , 曼陀罗叶
Petasites japonicus (Sieb.et Zucc.) Schmidt, rhizome of Japanese Butterbur, 蜂斗菜
Vigna sinensis (L.) Savi, Cowpea, 豇豆
Rana nigromaculata Hallowell, Pond green frog, 青蛙
Saurida tumbil (Blochet Schneider), meat, 蛇鲻
Phallus industiatus, fungus, 竹荪
Nicotiana tabacum L., leaf of Common Tobacco, 烟草
Areca catechu L., Areca seed, 槟榔
Gallus gallus domesticus Brisson, Membrane of chicken gizzard, 鸡内金
Euphorbia kansui Liou, root of Gansui, 甘遂
Amaranthus ascendens Loisel., all grass and root, 野苋菜
Allium cepa L., bulb of Common Onion, 洋葱
Drymaria cordata (L.) Willd., all-grass of West Indian chickweed, 荷莲豆菜
Luffa acutangula Roxb., Seed of Towel Gourd, 丝瓜子
Delongix regia (Boj.) Raf., twig and leaf of Flamboyantree, 凤凰木
Rosa roxburghii Tratt. f. normalis Rehd. et Wils., fruit of Single Roxburgh Rose, 刺梨
Allium fistulosum L., Fistular Onion bulb, 葱白
Phellinus igniarius (L.ex Fr.) Quel., fruiting body, 桑黄
Chimonanthus praecox (L.) Link, flower of Wintersweet, 蜡梅花
Stizolobium capitatum (Sweet) Kuntze, seed of Capitalteflower Velvetbean., 黎豆
Ulva lactuca L., algae, 石莼

Abroma angusta, root and bark, 昂天莲
Bubalus bubalis L., Buffalo horn, 水牛角
Pleurotus ostreatus (Jacg:Fr.)Kummer, mushroom, 侧耳
Juncus effusus L., Rush , 灯心草
Eclipta prostrata L., all-grass of Yerbadetajo, 墨旱莲
Thalictrum simplex L. var. brevipes Hara., root of Shortstalk slimtop Meadowrue, 硬水黄连
Glechoma Longituba (Nakai) Kupr., all-grass of Longtube Ground Ivy, 金钱草
Panax ginseng C.A.Mey., Ginseng root, 人参
Eucalyptus globulus Labill., leaf, 按叶
Cinchonaledgeriana(Howard)MoensetTrim, bark, 金鸡勒
Nardostachys jatamansi DC., rhizome of Spoonleaf Nardostachs, 甘松
Colysis hemionitidea (Wall.) Presl., leaf, 断线蕨
Enteromorpha Linza (L.) Ag., algae, 干苔
Melodinus fusiformis Champ.ex Benth., fruit of Fusiform Melodinus, 尖山橙
Thalictrum faberiulbr., root and rhizome of Faber Meadowrue, 大叶马尾连
Agrimonia pilosa Ledeb., root, 龙芽草根
Dryobalanops aromatica Gaertn.f., resin, 龙脑膏香
Scutellaria indica L., all-grass of Indian skullcup, 韩信草
Solanum melongena L., fruit of Garden Eggplant, 茄子
Abies nephrolepis, leaf and bark, 臭冷杉
Oplopanax elatus (Nakai) Nakai, root of Tall Oplopanax, 刺人参
Pteris fauriei Heiro., leaf, 金钗凤尾蕨
Viscum coloratum (Komar.) Nakai, wig, 槲寄生
Aloe vera L., Aloe, 芦荟
Trichosanthes kirilowii Maxim, Snakegourd peel, 栝楼皮
Carpesium cernuum L., all-grass of Drooping carpesium, 挖耳草
Artemisia lactiflora Wall., all-grass of Ghostplant Wormwood, 鸭脚艾
Rehmannia glutinosa Libosch., rhizome of Adhesive Rehmannia, 干地黄
Gekko gecko (L.), Red-spotted lizard, 蛤蚧
Hypophthalmichthys molitrix (Cuv.et Val.), Silver carp, 鲢鱼
Rhododendron mucronatum G.Don, leaf or root of Snow Azalea, 白花映山红
Aconitum carmichaeli Debx., Prepared lateral root of Common Monkshood., 附子
Ginkgo biloba L., Ginkgo leaf, 白果叶
Homo, urine, 人中白
Lentinus lepideus Fr., fruiting body, 豹皮菇

Astragalus complanatus R. Brown, seed, 沙苑蒺藜
Melilotus suaveolens Ledeb., all grass, 白花辟汗草
Arachis hypogaea L., seed skin, 花生衣
Biota orientalis (L.) Endl., leafy twigs of Chinese Arborvitae, 侧柏叶
Cordyceps sinensis (Bark.). Sacc., Chinese Caterpillar fungus, 冬虫夏草
Tinomiscium tonkinense Gagenp., root of Tonkin Tinomiscium, 大叶藤
Matricaria chamomilla L., flower of Mayweed, 母菊
Luffa acutangula Roxb., leaf of Towel Gourd, 丝瓜叶
Peganum harmala L., all-grass of Common peganum, 骆驼蓬
Argemone mexicana L., all grass, 蓟罂粟
Acanthopanax senticosus (Rupr.et Maxim.) Harms, root of Manyprickle Acanthopanax, 刺五加
Raphanus sativus L., Radish root, 莱菔
Lucuma nervosa A.DC., fruit of Eggfruit, 鸡蛋果
Cordyceps hawkesii Gray, fungus, 亚香棒虫草
Citrullus lanatus (Thurb.) Mansfeld, Watermelon rind, 西瓜皮
Crocus sativus L., stigma, 番红花
Thalictrum atriplex Finet et Gagnep., root and rhizome, 西藏水黄连
Rhododoendron mucronulatum Turcz., leaf of Korean Rhododoendron, 迎山红
Chrysanthemum segetum L., stem and leaf of South chrysanthemum, 茼蒿
Phyllostachys nigra Munro var.henonis (Mitf) Stapf.ex Rendle, Henon Bamboo Juice, 竹沥
Sinilabeo decorus decorus (Peters), meat, 竹鱼
Panax ginseng C.A.Mey., Ginseng rhizome, 人参芦
Cyclophiops major, smooth green snake, 竹叶青
Rhapis excelsa (Thunb.) Henry ex Rehd., leaf, 棕竹
Notopterygium incisum Ting ex H.T.Chang, root and rhizome of incised Notopterygium, 羌活
Syringa oblata Lindl., root of Early Lilac, 紫丁香
Hemerocallis plicata Stapf., root of Foldleaf Daylily, 黄花菜
Hedysarum polybotrys Hand.-Mazz., root of Manyinflorescenced Sweetvetch., 红芪
Brassica juncea (L.) Czern.et Coss., tender Stem and leaf of India Mustard, 芥菜
Artemisia eriopoda Bunge., all grass, 南牡蒿
Inula helenium L., root of Elecampane Inula, 土木香
Hedysarum austrosibiricumb.Fedtsch., all grass, 新疆红芪
Wine, distiller’s grains, 酒糟
Trifolium pratente L., all-grass of Red clover, 红车轴草
Orchis latifolia L., all-grass of Marsh Orchis, 红门兰

Rubus adenophorus Rolfe, root, 红牛毛刺根
Meconopsis punicea Maxim., scape and fruit of Redflower Meconopsis, 红花绿绒蒿
Sophora japonica L., Jew's Ear on pagodatree, 槐耳
Brassica campestris L., seed of Bird Rape, 芸薹子
Camellia sinensis Kuntze, Tea leaf, 茶叶
Misgurnus anguillicaudatus (Cantor), Oriental weatherfish, 泥鳅
Phragmites communis Trin., Reed leaf, 芦叶
Artemisia selengensis Turcz.ex Bess., all grass, 蒌蒿
Eucommia ulmoides Oliv., Eucommia leaf., 杜仲叶
Mangifera indica Linn, leaf, 杧果叶
Mangifera indica Linn, kernel, 杧果核
Prunus armenica L.var.ansu Maxim., fruit of Apricot, 杏子
Taxillus levinei (Merr.) H. S. Kiu, stem, 锈毛钝果寄生
Myrica rubra (Lour.) Sieb.et Zucc., bark of Chinese Waxmyrtle, 杨梅树皮
Setaria italica (L.) Beauv., Millet sprout, 粟芽
Juglans regia L., Walnut meat, 胡桃仁
Arenicola cristata stimpson., worm, 海蚯蚓
Fomitopsis officinalis (Vill. : Fr.) Bond., mushroom, 苦白蹄
Allium sativum L., Crown's Treacle, 大蒜
Phaseolus radiatus L., Mung Bean, 绿豆
Litchi chinensis Sonn., Litchi seed, 荔枝核
Amaranthus spinosusL, all grass or root, 簕苋菜
Salicornia europaea L., all grass, 海蓬子
Dryopteris filix-mas (L.) Schott, rhizome, 欧绵马
Chondria armata (Kütz.) Okam, algae, 软骨藻
Oncorhynchus Keta (Walb.), whole fish, 大马哈鱼
Hyriopsis cumingii (Lea), Nacre, 珍珠母
Clematis florida Thunb., root of Cream clematis, 铁线莲
Calendula Officinalis L., flower, 金盏菊花
Diospyros kaki L.f., root of Persimmon, 柿根
Codiaeum variegatum (Linn), leaf, 洒金榕
Panax ginseng C.A.Mey., Ginseng Leaf, 人参叶
Camellia sinensis Kuntze, Tea fruit, 茶子
Purpura gradata Jonas, shell, 蓼螺
Juglans regia L., leaf of English Walnut, 胡桃叶

Abrus precatorius, root, 相思子根

Allium tuberosum Rottl.ex Spreng, root, 韭根

Agave sisalana Perrine, leaf of Sisal Hempplant, 剑麻

Camelus bactrianus L., Camel as food, 骆驼肉

Bombyx mori L., Silkworm egg, 原蚕子

Castanea mollissima Bl., leaf of Hairy Chestnut, 栗叶

Castanea mollissima Bl., bark of Hairy chesstnut, 栗树皮

Castanea mollissima Bl., root of Hairy chestnut, 栗树根

Corbicula fluminea (Muller), freshwater small clam, 蚬肉

Triglochin maritimum L., all-grass of Shore Podgrass, 海韭菜

Cordyceps liangshanensis Zang. Liu et Hu, fungus, 凉山虫草

Oryza sativa, sweet ferment rice, 酒酿

Herba Zosterae Marinae, all grass, 大叶藻

Phoca vitulina, oil, 海豹油

Tamarindus indica L., fruit of Tamarind, 酸角

Melilotus officinalis (L.) Desr., all grass, 黄零陵香

Thevetia peruviana (Pers.) K.Schum., leaf of Luckynut Thevetia, 黄花夹竹桃叶

Ovis ammon Linndeus, horn, 盘羊角

Stemmacantha carthamoides (Willd.) Dit-trich, root, 鹿草

Cervus elaphus L., Refuse of Deerhorn glue, 鹿角霜

Ranarugosa (schlegel)., toad, 粗皮蛙

Cocos nucifera L., juice and rind, 椰子

Rhapis excelsa (Thunb.) Henry ex Rehd., root, 棕竹根

Trachycarpusfortunei (Hook.) H. Wendl., root, 棕榈根

Trachycarpus fortunei H.Wendl., leaf of Fortune Windmillpalm., 棕榈叶

Kerria japonica (L.) DC., wig, 棣棠枝叶

Carica papaya L., Papaya Leaf, 番木瓜叶

Viole tricolor L. Var. hortensis DC., all-grass of Garden Pansy, 三色堇

Ricinus communis L., Castor leaf, 蓖麻叶

Apis cerana Fabr, Bee Propolis, 蜂胶

Punica granatum L., sour fruit of Pomegranate, 酸石榴

Potentilla anserina L., all grass, 蕨麻草

Rehmannia glutinosa (Gaertn.) Libosch., prepared rhizome of Adhesive Rehmannia, 熟地黄

Extract of beef, 霞天膏

Panax ginseng C.A.Mey., Ginseng fruit, 人参子

Panax ginseng C.A.Mey., Ginseng flower, 人参花
Euphorbia kansui Liou, root of Gansui, 甘遂

Table S8 PcG-interacting TCM medicinals

PcG proteins
Clematis armandii Franch., stem of Armand clematis, 川木通
Temnopleurus hardwickii (Gray), Sea urchin, 海胆
Ostrea gigas Thunberg, Oyster shell, 牡蛎
Clerodendron Serratum (L.) Spreng., all-grass of Amplexifolious Glorybower, 三台红花
Periophthalmus modestus, fish, 弹涂鱼
Saussurea inuolucrata Kar. Et Kir., whole plant with flower, 天山雪莲花
Ajuga bracteosa Wall. Ex Benth., all-grass of Manybractelole Bugle, 九味一枝蒿
Mucuna castanea Merr., stem, 黑血藤
Ainsliaea fragrans Champ., all-grass of Frangrant Ainsliaea, 金边兔耳
Podocarpus macrophyllus (Thunb.) D.Don var.maki Endl., seed and receptacle of Kusamaki, 罗汉松实
Eupatorium cannabinum L., all grass, 大麻叶佩兰
Ardisia crenata Sims., root of Coral Ardisia, 朱砂根
Selaginella tamariscina (Beauv.) Spring, all-grass of Tamariskoid spikemoss, 卷柏
Mallotus repandus (Willd.) Muell-Arg., root or Stem and leaf of Creeping Mallotus, 杠香藤
Phoenix dactylifera L., Date, 无漏子
Asparagus cochinchinensis (Lour.)Merr., root of Cochinchinese Asparagus, 天门冬
Fagopyrum tataricum (L.) Gaertn., root and rhizome, 苦荞头
Artemisia capillaris Thunb., seedling of Capillary Wormwood, 茵陈蒿
Hovenia dulcis Thunb., fruit or seed of Japanese Raisin Tree, 枳椇子
Carica papaya L., Papaya, 番木瓜
Arctium lappa L., achene of Great Burdock, 牛蒡子
Rhododendron micranthum Turcz., leaf or flower of Manchurian Rhododendron, 照山白
Imperata cylindrica Beauv.var.major (Nees) C.E.Hubb., rhizome of Lalang Grass, 白茅根
Agrimonia pilosa Ledeb., all-grass of Hairyvein Agrimonia, 仙鹤草
Equisetum arvense Linn., all grass, 问荆
Hypericum perforatum L., all-grass of Common St.John'swort, 贯叶连翘
Pachysandra terminalis Sieb.et Zucc., all-grass of Japanese Pachysandra, 雪山林
Codonopsis tubulosa Kom., root of Pilose Asiabell, 党参
Smilax lanceifolia var. opace A.DC., rhizome of Bluebead Greenbrier, 土茯苓
Lophantherum gracile Brongn., all-grass of Common Lophantherum, 淡竹叶
Ipomoea batatas (L.) Lam., Sweet potato, 番薯

Dioscorea opposita Thunb., rhizome of Common Yam, 山药
Atractylodes macrocephala Koidz., rhizome of Largehead Artractylodes, 白术
Rubus chingii Hu, fruit of Palmleaf Rasberry, 覆盆子
Camellia japonica L., flower of Japanese camellia, 山茶花
Polygonum bistorta L., rhizome of Bistort, 拳参
Hyptis suavedens Poit., stem and leaf of Wild.spikenard, 蛇百子
Polygonatum macropodium Turcz., rhizome of Fragrant Solomonseal, 玉竹
Prunus persica (L.) Batsch, Peach kernel., 桃仁
Bupleurum komarovianum Lincz., root of Siberian Thorowax, 柴胡
Scopolia acutangula C. Y. Wu et C. Chen, root or Seed of Angularcalyx Scopolia, 三分三
Pyrus pyrifolia (Burm.f.) Nakai, fruit of Bretschneider pear, 梨
Averrhoa carambola L., fruit of Common Averrhoa, 阳桃
Lycoris radiata (L'Herit.) Herb, bulb of Shorttube Lycoris, 石蒜
Rhus succedanea L., root and leaf and bark and fruit, 野漆树
Angelica sinensis (Oliv) Diels., root of Chinese Angelica, 当归
Lantana camara L., Leaf of Common Lantana, 五色梅
Viburnum cylindricum Buch. Ham. Ex D. Don, leaf or bark, 水红木叶
Lysimachia clethroides Duby, root or all-grass of Clethra Loosestrife, 珍珠菜
Ipomoea digitata L., root or leaf of Fingerleaf Moningglory, 藤商陆
Pteridium aquilinum (L.) Kuhn var.latiusculum (Desv.) Underw, all-grass of Eastern Bracken Fern, 蕨
Rubia cordifolia L., root of India Madder, 茜草
Polygonatum macropodium Turcz., rhizome of Siberian Solomonseal., 黄精
Ilex pubescens Hook.et Arn., root of Pubescent Holly, 毛冬青
Costus speciosus (Koenig) Smith, rhizome of Canereed Spiralflag., 樟柳头
Litsea glutinosa (Lour) C.B.Bob., root or bark of Gluey Litse, 残槁蕴
Lamium barbatum Sieb. et Zucc., all-grass of Barbate Deadnettle, 野芝麻
Viburnum sargentii Koehne, twig and leaf of Sargent Craneberrybush., 鸡树条
Quisqualis indica L., fruit of Rangooncreeper, 使君子
Schisandra spenanthera Rehd.et Wils., fruit of Chinese Magnoliavine, 五味子
Phyllanthus urinaria L., whole plant, 叶下珠
Polygonum cuspidatum Sieb.et Zucc., rhizome of Giant Knotweed, 虎杖
Cornus officinalis Sieb. et Zucc., fruit of Asiatic Cornelian cherry, 山茱萸
Scutellaria rehderiana Diels, root of Baikal skullcap, 黄芩
Ampelopsis brevipedunculata (Maxim.) Trautv., root and bark, 蛇白蔹
Platycarya strobilacea Sieb.et Zucc., Leaf of Roundfruit Dyetree, 化香树叶

Dysosma pleiantha (Hance) Woodson, rhizome and root of Sixangular Dysosma, 八角莲
Astilboides tabularis (Hemsl.) Engl., all grass, 大叶子
Anisodus tanguticus (Maxim) Pascher, root or Seed of Tangut Anisodus, 藏茄
Sorbaria sorbifolia (L.) A. Brown, stem bark and wig, 珍珠梅
Glochidion macrophyllum Benth., fruit or leaf of Largeleaf Glochidion, 艾胶算盘子
Euscaphis japonica (Thunb.) Dippel, fruit or seed, 野鸦椿子
Polygala japonica Houtt., all-grass of Japanese Milkwort, 瓜子金
Bidens paruiflora Willd., all grass, 小鬼钗
Vitex negundo L., fruit, 黄荆子
Changium smyrnioides Wolff, root of Medicinal Changium, 明党参
Daucus carota L., all-grass of Wild Carrot, 鹤虱风
Scabiosa comosa Fisch.ex Roem .et Schult., flower of Narrowleaf Scabious, 蒙古山萝卜
Micromelum integerrimum (Buch.-ham.) Roem., root of Entire Micromelum, 小芸木
Rehmannia glutinosa (Gaertn.) Libosch., fresh rhizome of Adhesive Rehmannia, 鲜地黄
Prunella vulgaris L., fuit -spike of Common Selfheal, 夏枯草
Rhododendron dauricum L., leaf of Dahurian Rhododendron, 满山红
Vitis vinifera L., fruit of European Grape, 葡萄
Sophora flavescens Ait., root of Lightyellow sophora, 苦参
Rhodomyrtus tomentosa (Ait.) Hassk., leaf, 山稔叶
Crocosmia crocosmiflora (Nichols.) N. E. Br., bulb stem, 雄黄兰
Machilus thunbergii, bark, 红楠皮
Sapium discolor (Champ. ex Benth.) Muell.-Arg., leaf, 山乌桕叶
Lycium chinense Mill.etc., root-bark of Chinese Wolferry, 地骨皮
Millettia reticulata Benth., Stem of Leatherleaf Millettia, 昆明鸡血藤
Polygonum chinense L., all-grass of Chinese Knotweed, 火炭母草
Pyrola rotundifolia L. subsp. chinensis H.Andres, , 鹿衔草
Gaultheria cumingiana, leaf, 满山香
Murraya exotica L., Leaf and twig of Common Jasminorange, 九里香
Boenninghausenia sessilicarpa Levl., all-grass of Sessilefruit chinaure, 石椒草
Ocimum basilicum L., all-grass of Basilic Basil, 罗勒
Coriolus versicolor (Lr.) Quel, Polystictus versicolor (L.) Fr., 云芝
Oroxylum indicum (L.) Vent., seed of Indian Trumpetflower, 木蝴蝶
Lonicera japonica Thunb., Honeysuckle Stem, 忍冬藤
Lythrum salicaria L., all-grass of Spiked loosestrife, 千屈菜
Euphorbia humifusa Willd., all-grass of Humifuse Euphorbia, 地锦草

Artemisia anomala S.Moore, all-grass of Seleng Wormwood., 刘寄奴
Bischofia javanica Bl., root of Java Bishopwood, 秋枫木
Rhododendron capitatum Maxim., flower and leaf, 黑香柴
Vicia faba L., Broad bean, 蚕豆
Polygonum hydropiper L., all-grass of Red-knees, 水蓼
Phellodendron chinense Schneid., bark of Amur.corktree, 黄柏
Pyrus pashia Buch.-Ham.ex D. Don, bark, 川梨茎皮
Taraxacum platypecidum Diels., all-grass of Mongolian Dandelion, 蒲公英
Oxytropis glabra DC., all-grass of Glabrous Crazyweed, 醉马草
Ageratum Comyzoides L., all-grass of Tropic Ageratum, 胜红蓟
Pedilanthus tithymaloides, whole plant, 扭曲草
Panax quinquefolium L., American Ginseng, 西洋参
Euonymus sacrosancta Koidz, stem and root, 东北卫矛
Narcissus tazetta L. var. chinensis Roem., flower of Chinese Narcissus, 水仙花
Acer ginnala Maxim., bud, 桑芽
Patrinia villosa Juss., all-grass of Dahurian Patrinia, 败酱
Setaria italica (L.) Beauv., seed of Foxtail Millet, 粟米
Semen Dolichoris Album, White Hyacinth Bean, 白扁豆
Lutra lutra L., Otter liver, 獭肝
Fraxinus rhynchophylla Hance, bark of Bunge Ash., 秦皮
Oenanthe javanica (Bl.) DC., all-grass of Javan Waterdropwort, 水芹
Emmenopterys Henryi Oliv., root and bark, 香果树
Selaginella nipponica Franch.et Sav., whole plant, 小地柏
Galium aparine L. var. tenerum (Gren. et Godr.) Reichb., whole plant, 八仙草
Cassia tora L., seed of Sickle Senna, 决明子
Saussurea involucrata Kar. et Kir., all-grass of Lanatehead Sausuraea, 雪莲花
Phyllanthus amarus Schum. & Thonn., leaf, 小返魂
Lycopersicon esculentum Mill., Tomato, 番茄
Lycopus lucidus Turcz., stem and leaf of Shiny Bugleweed, 泽兰
Cyrtomium fortunei J. Smith, rhizome, 小贯众
Duchesnea indica (Andr.) Focke., all-grass of Indian Mockstrawberry, 蛇莓
Anethum graveolens L., Dill, 莳萝子
Ruta graveolens L., all-grass of Common Rue, 臭草
Bidens tripartita L., all-grass of Bur Bggarticke, 狼把草
Atropanthe sinensis (Hemsl.) Pascher, root, 天蓬子根

Jasminum humile L., leaf, 败火草
Conocephalum conicum(L.) Dumort., whole plant, 蛇地钱
Vicia hirsuta (L.)S.F. Gray, all-grass of Pigeon vetch, 小巢菜
Ficus microcarpa L., leaf of Smallfruit Fig., 榕树叶
Aeluropus pungens (M. Bieb.) C. Koch, leaf, 小獐毛
Asterina pectinifera Miller et Troschel, Petrel, 海燕
Polypodium vulgare L.；Polypodium virginianum L., rhizome, 多足蕨
Lespedeza formosa (Vogel) Koehne, whole plant, 马扫帚
Rhododendron capitatum Maxim., twig and Leaf of Capitate Rhododendron, 小叶杜鹃
Apocynum venetum L., all-grass of Dogbane, 罗布麻
Apis mellifera, bee, 蜂药
Adiantum davidii Franch., all-grass of Maidenhair, 猪鬃草
Cynoglossum amabile stapf et Drumm., all-grass of Chinese Forgetmenot, 狗屎花
Wahlenbergia marginata (Thunb.) A.DC., root of Marginate Rockbell., 兰花参
Lindernia crustacea (L.) F.Muell., all-grass of Brittle Falsepimpernel, 母草
Melilotus suaveolens Ledeb., all-grass of Daghestan Sweetclover, 辟汗草
Rhododendron seniavinii Maxim., leaf, 满山白
Euphorbia hirta L., all-grass of Garden Euphorbia, 大飞扬草
Spinacia oleracea L., all-grass of Spinach, 菠菜
Consolida ajacis (L.) Schur., root of Rocket consolida, 飞燕草
Salsola collina Pall., all-grass of Common Russianthistle, 猪毛菜
Passiflora caerulea L., all-grass of Passionflower, 西番莲
Portulaca oleracea L., all-grass of Purslane, 马齿苋
Solanum xanthocarpum Schrad.et Wendl., fruit of Yellowfruit Nightshade, 黄果茄
Monomorium pharaonis L., ant, 蚂蚁
Cervus nippon Temminck, Pilose Anter, 鹿茸
Plantago asiatica, leaf, 车前草
Vicia sativa L., all-grass of Common vetch, 大巢菜
Swainsonia salsula Taubert, all grass, 苦马豆
Melochia corchorifolia L., stem and leaf, 木达地黄
Euphorbia helioscopia L., all-grass of Sun Euphorbia, 泽漆
Equisetum pratense Ehrh., all grass, 草问荆
Artemisia japonica Thunb., all-grass of Japanese Wormwood., 牡蒿
Lagerstroemia subcostata Koehne, flower or root of Southern Crapemyrtle, 拘那花
Brasenia schreberi J.F.Gmel., Watershield, 莼

Caltha palustris L., root and leaf, 驴蹄草
Anodonta woodiana (Lea), Pearl, 珍珠
Cucumis sativus L., Cucumber, 黄瓜
Astragalus membranaceus (Fisch.) Bge. Var. Monghol, Radix Astragali, 黄芪
Sapium sebiferum (L.) Roxb., root and bark, 乌桕木根皮
Sophora japonica L., pod of Japanese Pagodatree, 槐角
chloranthus japonicus Sieb., all-grass of Japanese chloranthus, 银线草
Trichosanthes japonica Regel, root of oneflower snakegourd, 天花粉
Nandina domestica Thunb., leaf of Common Nandina, 南天竹叶
Bambusa textilis Mc-Clure, CONCRETIO SILICEA BAMBUSAE, 竹黄
Bryophyllum pinnatum (L.f.) Oken, all-grass of Air-plant, 落地生根
Physalis peruviana L., all-grass of Capegcoseberry, 灯笼草
Arisaema fargesii Buchet, tuber of Farges Jackinthepulpit, 螃蟹七
Erodium stephanianum Willd., all-grass of Common Heron's bill, 老鹳草
Aucuba chinensis Benth., Leaf of Chinese Aucuba, 天脚板
Tetrapanax papyriferus (Hook.) K.Koch., pith of Ricepaperplant, 通草
Malus pumila Mill., Apple, 苹果
Cuscuta chinensis Lam., seed, 菟丝子
Elaeagnus umbellata Thunb., root and leaf and fruit, 牛奶子
Citrullus lanatus (Thurb.) Mansfeld, Pulp of a Watermelon, 西瓜
Equisetum hiemale L., horsetail, 木贼
Insect gall, Nutgalls, 没食子
Ziziphus jujuba Mill, Chinese date, 大枣
Pinellia ternata (Thunb.) Breit., tuber of Pinellia, 半夏
Eriocheir sinensis H.Milne-Edwards, Carb, 蟹
Artemisia subdigitata Mattf., all-grass of Subdigitate wormwood, 牛尾蒿
Veronica anagallis-aquatca L, all grass, 水苦荬
Uncaria hirsuta Havil, Gambirplant stem with hooks., 钩藤
Euphoria longan (Lour). Steud., Longan aril, 龙眼肉
Auricularia auricula (L.ex Hook.) Underw., Jew's Ear, 木耳
Phaseolus vulgaris, bean, 菜豆
Glycyrrhiza uralensis Fisch., root of Glabrousfruit Licorice, 甘草
Hippophae rhamnoides Linn., fruit, 沙棘
Dioscorea alata L., rhizome of Winged yam, 毛薯
Ganoderma lucidum, Lingzhi mushroom, 灵芝

Bruguiera gymnorrhiza (L.) Lam., bark, 红树皮
Aegie marmelos (L.) Correa, fruit, 硬皮橘
Alnus japonica (Thunb) Steud., twig and leaf of Japanese Alder, 赤杨
Hamamelis mollis Oliv., root of Chinese witchhazel, 金缕梅
Ganoderma applanatum (Pers.ex Wallr.) Pat., fruiting body, 树舌
Gossampinus malabarica (DC.) Merr., flower of Common Bombax, 木棉花
Lanminaria japonica Aresch., Kelp, 昆布
Abutilon indicum (L.) Sweet, all-grass of Indian Abutilon, 磨盘草
Caragana frutex (L.) Koch, flower of Russian peashrub, 木锦鸡儿
Potentilla fruticosa L., leaf, 金老梅叶
Artemisia argyi Levl. et Vant., leaf of Argy Wormwood, 艾叶
Antidesma bunius (L.) Spr., root or leaf of Bignay chinalaurel, 五月茶
Phaseolus lunatus L., Sieve Bean., 金甲豆
Salacia prinoides (Willd.) DC, root, 桫拉木
Sageretia thea (Osbeck) Johnst., twig and leaf of Hedge Sageratia, 雀梅藤
Rhus chinensis Mill, root, 盐肤木根
Illicium Verum Hook.f., fruit of Truestar Anisetree, 八角茴香
Penaeus orientalis Kish., lobster, 对虾
Cabomba caroliniana, Axle Algae, 鱼草
Urtica macrorrhiza Hand.-Mazz., whole plant, 青活麻
Bidens pilosa L., all grass, 盲肠草
Campylotropis hirtella (Franch) schindl., root of Hairy Clovershrub, 大红袍
Tribulus terrestris L., fruit, 刺蒺藜
Lycium dasystemum pojark., fruit of Hairystamen Wolfberry, 枸杞子
Ceohtopodium subulatum (Franch) Beauv, all grass, 苦艾
Apis mellifera, Honey, 蜂蜜
Anguilla japonica Temminck et Schlegel, Japanese eel, 鳗鲡鱼
Clerodendrum inerme (L.) Gaertn., twig and leaf of Unarmed Glorybower, 水胡满
Bubalus bubalis L., Ox blood, 牛血
Phyllanthus emblica L., fruit of Emblic leafflower, 余甘子
Lygodium flexuosum (L.) Sw., all-grass of Flexuose Climbing Fern, 牛抄藤
Xanthium sibircium Patr. ex Widd., fruit of Siberian Cocklebur, 苍耳子
Lawsonia inermis L., Henna leaf, 指甲花叶
Colocasia esculenta (L.) Schott., tuber of Dasheen, 芋头
Lepisma saccharina L., silverfish, 衣鱼

Inula japonica Thunb., all-grass of Japanese Inula, 金沸草
Eucalyptus citriodora, leaf, 柠檬桉叶
Lithospermum arvense Linn., all grass, 田紫草
Ilex pubescens Hook.et Arn., Leaf of Pubescent Holly, 毛冬青叶
Heteropogon contortus (L.) Beauv., root or all-grass of Contorted Tanglehed, 地筋
Ocimum basilicum L., fruit, 罗勒子
Evodia rutaecarpa (Juss.) Benth. var. officinalis (Dode) Huang., fruit of Medicinal Evodia, 吴茱萸
Solidago virgaurea L., flower, 新疆一枝黄花
Rana nigromaculata Hallowell, Pond green frog, 青蛙
Adiantum monochlamys Eaton, all grass, 石长生
Premna puberula Pamp., leaf of Puberulent Premna, 斑鸠占
Thesium chinensis Turcz., all-grass of Chinese Bastardtoadflax., 百蕊草
Nicotiana tabacum L., leaf of Common Tobacco, 烟草
Euphorbia kansui Liou, root of Gansui, 甘遂
Saposhnikovia divaricata (Turcz.) Schischk., root of Divaricate Saposhnikovia, 防风
Litchi chinensis Sonn., Lychee Nut, 荔枝
Glycine max.(L.) Merr., black seed of Soybean., 黑大豆
Luffa acutangula Roxb., Seed of Towel Gourd, 丝瓜子
Sapium sebiferum (L.) Roxb., leaf, 乌桕叶
Opuntia dillenii (Ker-Gawl.) Haw., root and stem of Cholla, 仙人掌
Impatientis balsamina L., root of Garden Balsam., 凤仙根
Impatiens balsamina L., stem, 凤仙透骨草
Delongix regia (Boj.) Raf., twig and leaf of Flamboyantree, 凤凰木
Fragaria ananassa Duchesne, stawberry, 草莓
Rosa roxburghii Tratt. f. normalis Rehd. et Wils., fruit of Single Roxburgh Rose, 刺梨
Artemisia apiacea Hance, all-grass of Celery Wormwood., 青蒿
Uncaria gambier Roxb., extract of wig, 方儿茶
Zinnia elegans Jacq., all-grass of Youth-and-old-age, 百日草
Helicteres isora L., root of Tortedfruit Screwtree, 火索麻
Viburnum cordifolium Wall. Et D C., root, 心叶荚蒾根
Pharbitis purpurea (L.) Voight, Pharbitis seed, 牵牛子
Morinda officinalis How, root of Medicinal Indianmulberry, 巴戟天
Marchantia polymorpha L., all grass, 地钱
Agaricus campestris L.ex Fr., Meadow mushroom Mushroom, 蘑菇
Adenanthera pavonina L., seed of Sandal Beadtree, 海红豆

Acorus calamus L., rhizome, 水菖蒲
Corchorus capsularis L., Leaf of Roundpod Jute, 黄麻叶
Silybum marianum (L.) Gaertn., all-grass of St. Marys, 水飞蓟
Pleurotus ostreatus (Jacg:Fr.)Kummer, mushroom, 侧耳
Cyperus rotundus L., rhizome of Nutgrass Galingale, 香附
Saurauia tristyla DC.var.oldhamii Hemsl., root of Taiwan saurauia, 水冬瓜
Commelina communis L., all-grass of Common Dayflower, 鸭跖草
Juncus effusus L., Rush , 灯心草
Ammannia baccifera L., all-grass of Common Ammannia, 水苋菜
Menyanthes trifolia L., all-grass of Bogbean, 睡菜
Phragmites communis Trin., Reed rhizome, 芦根
Glechoma Longituba (Nakai) Kupr., all-grass of Longtube Ground Ivy, 金钱草
Panax ginseng C.A.Mey., Ginseng root, 人参
Eucalyptus globulus Labill., leaf, 按叶
Jasminum sambac (L.) Aiton, leaf of Arabian Jasmine, 茉莉叶
Canarium album (Lour.) Raeusch., fruit of White canarium, 橄榄
Saccharum sinensis Roxb., Sugarcane, 甘蔗
Cucumis meio L., Muskmelon seed, 甜瓜子
Polygonatum verticillatum (L.) All., rhizome of Whorledleaf Solomonseal., 羊角参
Sinocrassula indica (Decne) Berger, all-grass of Indian Sinocrassula, 石莲
Enteromorpha Linza (L.) Ag., algae, 干苔
Asparagus officinalis L., root, 石刁柏
Selaginella doederleinii Hieron., all grass, 石上柏
Psilotum nudum (L.) Eriseb., all-grass of Nude Fern, 石刷把
Adhatoda vasica Nees., twig and leaf of Malabarnut, 大驳骨
Poa sphondylodes Trin. ex Bunge, all grass, 硬质早熟禾
Smilax scobinicaulis C. H. Wright, rhizome and root, 铁丝灵仙
Euphoria longan (Lour). Steud., Longan leaf, 龙眼叶
Agrimonia pilosa Ledeb., root, 龙芽草根
Artemisia sphaerocephala Krasch., all-grass of Roundhead Wormwood., 白沙蒿
Abies nephrolepis, leaf and bark, 臭冷杉
Taxus cuspidata Sieb. et Zucc., twig and leaf of Japanese Yew, 紫杉
Tripterygium regeli Sprague et Tak., root of Regel Threewingnut, 东北雷公藤
Cucurbita moschata (Duch.) Poiret, furit of Cushaw, 南瓜
Glehnia littoralis F.Schmidt ex Miq., root of Coastal Glehnia, 北沙参

Dictamnus dasycarpus Turcz., root and bark, 白鲜皮
Aloe vera L., Aloe, 芦荟
Rubus crataegifolius Bunge, root, 托盘
Beta vugaris L. Var. Cruenta Alef., root, 萘菜根
Kalanchoe spathulata DC., all grass, 匙叶伽蓝菜
Dichirostachys glomerata (Forsk) Chiov., extract of wig, 柏勒树儿茶
Mesona chinensis Benth., all-grass of Chinese Mesona, 凉粉草
Oryza Sativa L., Rice, 粳米
Salix alba L., leaf of Cricket-bat willow, 白柳
Sophora japonica L., flower bud of Japanese Pagodatree, 槐花
Duranta repens L., fruit of Creeping skyflower, 假连翘
Trifolium repens L., all-grass of White Clover, 三消草
Lagetstroemia speciosa, root and leaf, 大叶紫薇
Fluggea virosa (Roxb. ex Willd.) Baill., whole plant, 白饭树
Benincasa hispida (Thunb.) Cogn., exocarp of Chinese Waxgourd, 冬瓜皮
Bacopa monnieri (L.) Wettst., all-grass of Coastal Waterhyssop., 白花猪母菜
Xanthium sibircium Patr. ex Widd., fruit of Siberian Cocklebur, 苍耳
Euphorbia tirucalli, all grass, 绿玉树
Morus alba L., root-bark of White Mulberry, 桑白皮
Aster subulatus Michx., all-grass of Annual Saltmarsh Aster, 瑞连草
Astragalus complanatus R. Brown, seed, 沙苑蒺藜
Melilotus suaveolens Ledeb., all grass, 白花辟汗草
Arachis hypogaea L., seed skin, 花生衣
Biota orientalis (L.) Endl., leafy twigs of Chinese Arborvitae, 侧柏叶
Erycibe obtusifolia Benth, root or stem of Obtuseleaf Erycibe, 丁公藤
Pisum sativum L., Pea, 豌豆
Lancea tibetica Hook.f.et Thoms., all-grass of Tibet Lancea, 兰石草
Pterospermum heterophyllum Hance, leaf of Heterophyllous Wingseedtree, 半枫荷叶
Armillariella tabescens (Scop .ex Fr.) Sing, fungus, 亮菌
Thevetia peruviana (Pers.) K.Schum., seed of Luckynut Thevetia, 黄花夹竹桃
Matricaria chamomilla L., flower of Mayweed, 母菊
Quercus acutissima Carr., fruit of Sawtooth Oak, 橡实
Psoralea corylifolia L., fruit of Malaytea scurfpea, 补骨脂
Polyporus umbellataus (Pers.) Fries, Umbellate pore Fungus, 猪苓
Raphanus sativus L., Radish root, 莱菔

Selaginella sanguinolenta (L.) Spring, all grass, 地柏树
Frullania tamarisci (L.), moss, 串珠耳叶苔
Lucuma nervosa A.DC., fruit of Eggfruit, 鸡蛋果
Crocus sativus L., stigma, 番红花
Hipeastrum vittalum, stem, 朱顶红
panax pseudoginseng Wall. Var. Japonicus (C.A.Mey) Hoo & Tseng, rhiome of Largelaef Japanese Ginseng, 珠儿参
Momordica grosvenori Swingle, fruit of Grosvenor Momordica, 罗汉果
Macrothelypteris oligophlebia (Bak.) Ching, rhizome, 金鸡尾巴草根
Chrysanthemum segetum L., stem and leaf of South chrysanthemum, 茼蒿
Doryopteris concolor (Langsd. Et Fisch.) Kuhn, all grass, 黑心蕨
Cicer arietinum L., seed of Gram Chickpea, 回回豆
Juniperus rigida Sied .et Zucc., fruit, 杜松
Cinnamomum tamala (Buch.-Ham.) Nees et Eberm., bark or leaf, 柴桂
Phyllostachys nigra Munro var.henonis (Mitf) Stapf.ex Rendle, Henon Bamboo Juice, 竹沥
Notopterygium incisum Ting ex H.T.Chang, root and rhizome of incised Notopterygium, 羌活
Eupatorium chinense L., root, 华泽兰
Hemerocallis plicata Stapf., root of Foldleaf Daylily, 黄花菜
Lithocarpus polystachhyus (Wall.) Rehd., leaf of Manyspike Tanoak, 多穗石柯叶
Adonis amurensis Regel et Radde, all grass, 冰凉花
Capra hircus Linnaeus, Goat or sheep blood, 羊血
Clitoria ternatea L., leaf of Asian pigeonwings, 蝴蝶花豆
Cleredendrum cwtophyllum Turcz., stem and leaf, 大青
Bauhinia variegata L., bark, 羊蹄甲树皮
Heteropappus altaicus (Willd.) Novopokr., root of Tatarian Aster, 紫菀
Orgza sativa L., Rice bran, 米皮糠
Hydnocarpus anthelmintica pier., Chaulmoogra seed, 大风子
Angelica dahurica, root, 大活
Carthamus tinctorius L., Safflower, 红花
Castanopsis hichelii A.Camus, seed of Hickel Evergreenchinkapin, 红橡木子
Orchis latifolia L., all-grass of Marsh Orchis, 红门兰
Sabian chinensis (L.) Antoine, leaf of Chinese Juniper, 桧叶
Hypserpa nitida Miers, all-grass of Shining Hypserpa, 夜花藤
Calophyllum inophyllum L., root of Kalofium, 红厚壳
Woodfordia fruticosa (L.) Kurz., flower of Shrubby woodfordia, 虾子花
Rhus punjabensis Stew.var.Sinica(Diels)Rehd.et Wils., root, 红麸杨根

Tulipa gesneriana L., flower of Common Tulip, 郁金香
Scylla serrata, crab, 青蟹
Sophora japonica L., Jew's Ear on pagodatree, 槐耳
Eucalyptus globulus Labill., leaf and fruit, 洋草果
Boehmeria tricuspis (Hance) Makino, leaf or root of Tricuspidate Falsenettle, 赤麻
Coriandrum sativum L., all-grass of Coriander, 胡荽
Coriandrum sativum L., fruit, 胡荽子
Camellia sinensis Kuntze, Tea leaf, 茶叶
Phragmites communis Trin., Reed leaf, 芦叶
Arudo donax L, Giantreed rhizome, 芦竹根
Canna edulis Ker, rhizome, 蕉芋
Folium Cycadis, leaf, 苏铁叶
Kleinhovia hospita Linn., leaf, 画头叶
Cunninghamia lanceolata (Lamb.) Hook., leaf of Chinese Fir, 杉叶
Mangifera indica Linn, bark, 杧果树皮
Prunus armenica L.var.ansu Maxim., fruit of Apricot, 杏子
Prunus armeniaca L., flower of Ansu Apricot, 杏花
Cantharellus cibarius Fr., fruiting body, 鸡油菌
Paeonia suffruticosa Andr., Tree Peony flower, 牡丹花
Rosa multiflora Thunb.var.carnea Thory, root and leaf, 十姊妹
Setaria italica (L.) Beauv., Millet sprout, 粟芽
Forsythia viridissima Lindl., root or leaf, 金钟花
Terminalia chebula Retz., fruit of Tomentose Terminalia, 诃子
Heteropappus altaicus (Willd.) Novopokr., all-grass of Altai Heteropappus, 阿尔泰紫菀
Gallus gallus domesticus Brisson, egg white, 鸡子白
Gallus gallus domesticus Brisson, Yolk, 鸡子黄
Millettia nitida, stem, 亮叶崖豆藤
Plumeria rubra L.var, acutifolia Bailey, flower of Mexican Frangipani, 鸡蛋花
Phaseolus radiatus L., Mung Bean, 绿豆
Rosa rugosa Thunb., Rose, 玫瑰花
Sophora alopecuroides L., all-grass of Foxtail-like Sophora, 苦豆子
Sophora alopecuroides L., seed, 苦豆草
Melia azedarach L., leaf, 苦楝叶
Millettia pachycarpa Benth., leaf, 苦擅叶
Imperata Cylindrica Beauv. var. major (Nees) C.E.Hubb., Leaf of LaLang Grass, 茅草叶

Equisetum sylvaticum L., all grass, 林问荆
liquidambar formosana Hance, leaf of Beautiful sweetgum, 枫香树叶
Euphorbia antiquorum L., whole plant, 霸王鞭
Viburnum dilatatum Thunb., stem and leaf of Linden Viburnum, 荚蒾
Levisticum officinale Koch, root of Garden Lovage, 欧当归
Clerodendranthus spicatus (Thunb.) C.Y.Wu., all-grass of Spicate clerodendranthus, 猫须草
Eragrostis ferruginea (Thunb.) Beauv., all-grass of Korean Lovegrass, 知风草
Lathyrus pratensis L., leaf, 牧地山黧豆
Collocalia esculenta L., Edible Bird's Nest, 燕窝
Cytisus scoparius Link, all-grass of Scotch Broom, 金雀儿
Calendula Officinalis L., flower, 金盏菊花
Humulus lupulus L., Hops, 啤酒花
Cimicifuga simplex Wormsk., rhizome of Kamchatka Bugbane, 野升麻
Camillia oleifera Abel, seed, 油茶子
Physochlaina physalioides (L.) G. Don, root, 泡囊草根
Panax ginseng C.A.Mey., Ginseng Leaf, 人参叶
Astragalus sinicu L., seed of Chinese Milkvetch., 紫云英子
Vaccinium bracteatum Thunb., leaf of Oriental Blueberry, 南烛叶
Cupressus funebris Endl., twig and leaf of Chinese Weeping cypress, 柏树叶
Diospyros kaki L.f., calyx and receptacle of a persimmon, 柿蒂
Diospyros kaki L.f., fruit of Persimmon, 柿子
Diospyros kaki L.f., leaf of Persimmon, 柿叶
Elsholtzia densa Benth., all grass, 咳嗽草
Woodfordia fruticosa (L.) Kurz, all grass, 虾子花叶
Thuja occidentalis L., leaf and wood, 香柏
Agave sisalana Perrine, leaf of Sisal Hempplant, 剑麻
Phytolacca acinosa Roxb, leaf, 美商陆叶
Morus alba L., Mulberry leaf, 桑叶
Prunus persica (L.) Batsch, Peach., 桃子
Prunus davidiana (Carr.) Franch., Peach leaf, 桃叶
Prunus davidiana (Carr.) Franch., Peach bark, 桃茎白皮
Prunus davidiana (Carr.) Franch., Peach blossom, 桃花
Castanea mollissima Bl., leaf of Hairy chestnut, 栗叶
Castanea mollissima Bl., bark of Hairy chesstnut, 栗树皮
Castanea mollissima Bl., root of Hairy chestnut, 栗树根

Psidium guajaua L., fruit, 番石榴果
Salvia glutinosa L., root, 胶质鼠尾草
Tamarindus indica L., fruit of Tamarind, 酸角
Morus alba L., Mulberry twig, 桑枝
Citrus tangerina Hort et Tanaka, Tangerine, 橘
Corchorus capsularis L., root of Roundpod Jute, 黄麻根
Catalpa ovata G.Don., wood, 梓木
Angelica koreana Maxim., root of Korean Angelica, 朝鲜当归
Lamium barbatum Sieb. et Zucc., root of Barbate Deadnettle, 野芝麻根
Glochidion puberum (L.) Hutch., leaf of Puberulous Glochidion, 算盘子叶
Stevia rebaudiana (Bertoni) Hemsl, leaf, 甜叶菊
Saccharum spontaneum L., rhizome of Wild Sweetcane, 甜根子草
Cannabis sativa L., root of Hemp Fimble, 麻根
Cocos nucifera L., juice and rind, 椰子
Lagerstroemia indica L., leaf of Common Crapemyrtle, 紫薇叶
Panicum miliaceum L., Broomcorn Millet root, 黍根
Annona reticulata L., leaf, 番荔枝叶
Psidium guajaua L., bark, 番石榴树皮
Viole tricolor L. Var. hortensis DC., all-grass of Garden Pansy, 三色堇
Ricinus communis L., Castor leaf, 蓖麻叶
Tribulus terrestris L., all-grass of Puncturevine Caltrop, 蒺藜苗
Punica granatum L., sour fruit of Pomegranate, 酸石榴
Potentilla anserina L., all grass, 蕨麻草
Citrus erythrosa Tanaka, Tangerine leaf, 橘叶
Murraya paniculata (L.) Ja, flower, 九里香花
Vernonia uolkameriifolia (Wall.) DC, whole plant, 大叶鸡菊花
Euonymus japonicus Thunb, leaf, 大叶黄杨叶
Caretta caretta (L.), Turtle blood, 蠵龟血
Fagopyrum cymosum (Trev.) Meisn., rhizome, 金荞麦

Table S9 DICER-interacting TCM medicinals

Drosha and Dicer
Angelica sinensis (Oliv) Diels., root of Chinese Angelica, 当归
Ligusticum chuanxiong Hort., rizome of Chuanxiong Ligusticum, 川芎
Ligustrum lucidum Ait., fruit of Glossy privet, 女贞子
Astragalus membranaceus (Fisch.) Bge. Var. Monghol, Radix Astragali, 黄芪
Ganoderma lucidum, Lingzhi mushroom, 灵芝
Medicago sativa L., all grass, 苜蓿
Lycium dasystemum pojark., fruit of Hairystamen Wolfberry, 枸杞子
Rana nigromaculata Hallowell, Pond green frog, 青蛙
Panax ginseng C.A.Mey., Ginseng root, 人参
Actinolite, 阳起石
Melilotus suaveolens Ledeb., all grass, 白花辟汗草
Peganum harmala L., all-grass of Common peganum, 骆驼蓬
Trifolium pratente L., all-grass of Red clover, 红车轴草
Misgurnus anguillicaudatus (Cantor), Oriental weatherfish, 泥鳅
Melilotus officinalis (L.) Desr., all grass, 黄零陵香
Stevia rebaudiana (Bertoni) Hemsl, leaf, 甜叶菊
Ranarugosa (schlegel)., toad, 粗皮蛙
